# The role of the plasmon in interfacial charge transfer

**DOI:** 10.1126/sciadv.adp3353

**Published:** 2024-07-05

**Authors:** Behnaz Ostovar, Stephen A. Lee, Arshad Mehmood, Kieran Farrell, Emily K. Searles, Briley Bourgeois, Wei-Yi Chiang, Anastasiia Misiura, Niklas Gross, Alexander Al-Zubeidi, Jennifer A. Dionne, Christy F. Landes, Martin Zanni, Benjamin G. Levine, Stephan Link

**Affiliations:** ^1^ Center for Adopting Flaws as Features, University of Illinois at Urbana-Champaign, Urbana, IL, USA.; ^2^ Department of Electrical and Computer Engineering, Rice University, Houston, TX, USA.; ^3^ Department of Chemistry, University of Illinois at Urbana-Champaign, Urbana, IL, USA.; ^4^ Department of Chemistry, Rice University, Houston, TX, USA.; ^5^ Institute for Advanced Computational Science, Stony Brook University, Stony Brook, NY, USA.; ^6^ Department of Chemistry, Stony Brook University, Stony Brook, NY, USA.; ^7^ Department of Chemistry, University of Wisconsin–Madison, Madison, WI, USA.; ^8^ Materials Science and Engineering, Stanford University, Stanford, CA, USA.; ^9^ Department of Radiology, Stanford University School of Medicine, Stanford, CA, USA.; ^10^ Department of Chemical and Biomolecular Engineering, University of Illinois at Urbana-Champaign, Urbana, IL, USA.; ^11^ Department of Chemical and Biomolecular Engineering, Rice University, Houston, TX, USA.; ^12^ Department of Electrical and Computer Engineering, University of Illinois at Urbana-Champaign, Urbana, IL, USA.

## Abstract

The lack of a detailed mechanistic understanding for plasmon-mediated charge transfer at metal-semiconductor interfaces severely limits the design of efficient photovoltaic and photocatalytic devices. A major remaining question is the relative contribution from indirect transfer of hot electrons generated by plasmon decay in the metal to the semiconductor compared to direct metal-to-semiconductor interfacial charge transfer. Here, we demonstrate an overall electron transfer efficiency of 44 ± 3% from gold nanorods to titanium oxide shells when excited on resonance. We prove that half of it originates from direct interfacial charge transfer mediated specifically by exciting the plasmon. We are able to distinguish between direct and indirect pathways through multimodal frequency-resolved approach measuring the homogeneous plasmon linewidth by single-particle scattering spectroscopy and time-resolved transient absorption spectroscopy with variable pump wavelengths. Our results signify that the direct plasmon-induced charge transfer pathway is a promising way to improve hot carrier extraction efficiency by circumventing metal intrinsic decay that results mainly in nonspecific heating.

## Teaser

Combined frequency- and time-resolved spectroscopy establishes mechanisms of charge transfer for plasmonic interfaces.

## INTRODUCTION

Plasmonic nanoparticles integrated with a semiconductor hold great promise for improving the efficiency of light-harvesting systems toward generating currents or driving chemical reactions with visible illumination (*1*–*4*). The localized surface plasmon resonance (LSPR), the collective oscillation of conducting electrons, offers intense, broadly tunable light absorption in metallic nanostructures (*5*–*7*). Simultaneously, LSPR decay generates a nonequilibrium distribution of energetic “hot” electrons and holes, which thermalize with the metal lattice on the few picosecond timescale (*7*–*11*). Efficiently collecting these energetic carriers before they thermalize is a substantial challenge, limiting the efficiency of potential plasmon-based light-harvesting applications (*3*). Nevertheless, plasmon-induced charge transfer at the metal-semiconductor interface has been used to facilitate hot carrier transfer to the semiconductor before thermalization with the metal phonon bath.

The collection of hot carriers by the metal nanostructure’s semiconductor interface is achieved by the formation of a Schottky barrier that assists in trapping the transferred hot electrons in the conduction band of the semiconductor (*2*, *3*, *12*–*20*)(*21*). This plasmon-induced electron transfer strategy effectively extends the lifetime of electrons in the conduction band of the semiconductor, making these heterostructures capable of advanced device architectures (*2*, *3*, *12*–*17*, *22*–*24*)(*21*). Furthermore, technologies based on metal-semiconductor heterostructures facilitate efficient utilization of visible light, circumventing the limitation of commonly used wide-bandgap semiconductors as the photon energy only needs to exceed the Schottky barrier and not their larger bandgap (*25*, *26*). Several devices based on plasmon-induced charge transfer have been developed, confirming the charge transfer process by producing a photocurrent or driving a chemical reaction. However, reported efficiencies vary greatly (*1*, *27*–*31*). A quantitative characterization of the mechanism underlying plasmon-induced charge transfer at the metal-semiconductor interface remains elusive (*9*, *26*). This lack of vital understanding hinders not only a rational interpretation of reported efficiencies but also the further development of photovoltaic devices and photocatalysts.

**Fig. 1.**
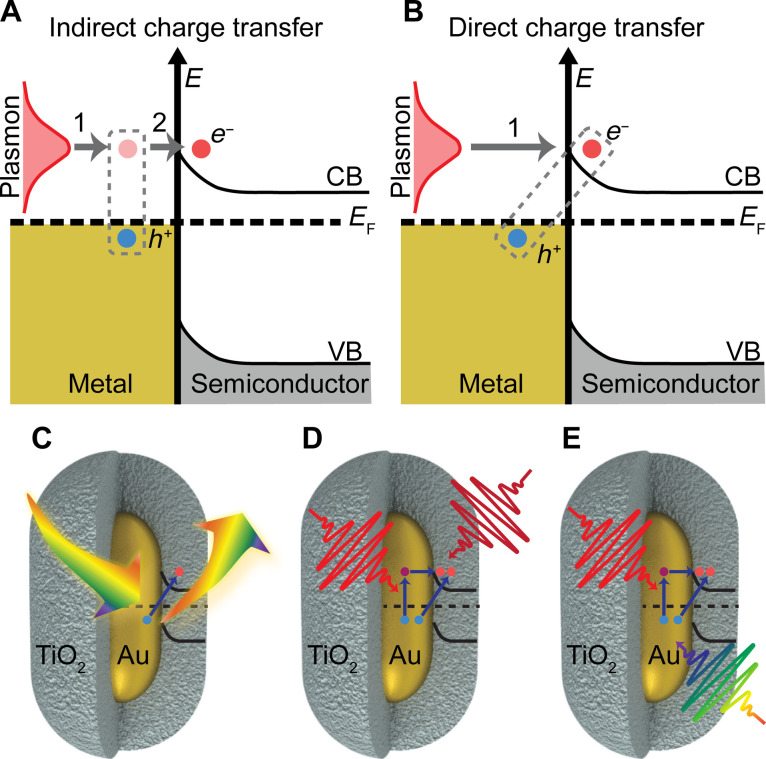
Distinguishing indirect and direct plasmon-induced charge transfer. (**A**) Indirect plasmon-induced charge transfer, in which plasmons decay by generating hot electron-hole pairs in the metal, followed by transfer of the hot electrons into the conduction band of the semiconductor. (**B**) Direct plasmon-induced charge transfer, in which the plasmon decays by excitation of an electron in the conduction band of the semiconductor and a hole inside the metal. The energies of the valance band (VB), conduction band (CB), and Fermi energy (*E*_F_) are displayed. Experimental design for (**C**) detecting direct plasmon-induced charge transfer through CID of plasmons for AuNRs@TiO_2_. (**D**) Transient absorption measurements using visible pump wavelengths that mediate electron transfer from Au to the conduction band of TiO_2_ through direct and indirect plasmon-induced charge transfer pathways as well as nonplasmon resonant excitation. IR and NIR probe wavelengths were used to study the dynamics of the injected electrons within the TiO_2_ conduction band; (**E**) visible broadband probe of dynamics of remaining hot carriers inside the Au.

Two pictures of plasmon-induced charge transfer at metal-semiconductor interfaces have emerged. One involves the following sequential steps: (i) hot electron-hole pair generation inside the metal, followed by (ii) transfer of hot carriers with energies higher than the Schottky barrier from the metal into the semiconductor (Fig. 1A) (*3*, *22*). The timescale of this indirect charge transfer process has been estimated to be around ~100 to 1000 fs using Fowler’s theory of ballistic transport (*14*, *32*). In contrast, a direct electron transfer pathway has also been proposed, involving the decay of a surface plasmon by direct excitation of an electron in the conduction band of the semiconductor and a hole in the metal (Fig. 1B) (*15*, *33*, *34*). This direct plasmon-induced charge transfer is similar to a long-neglected process known as chemical interface damping (CID) of plasmons (*35*, *36*). Theoretical models support this direct charge transfer pathway and predict an efficiency of 40% for the separation of electron-hole pairs at the metal-semiconductor interface with a timescale of ~10 to 20 fs (*37*). This large electron injection efficiency arises because the direct charge transfer pathway circumvents energy loss inside the metal by fast electron-electron scattering (~10 to 100 fs) (*38*, *39*) and electron-phonon coupling (~1 ps) (*40*), subsequently creating unwanted heating. An experimental distinction between indirect and direct pathways in metal-semiconductor heterostructures has so far not been established, mainly because the short pulses necessary to distinguish timescales of 10 fs (direct) and 100 fs (indirect) intrinsically cannot have the spectral resolution needed to selectively excite only the LSPR.

Here, we use a fundamentally different approach by combining time-resolved spectroscopy with frequency-resolved single-particle measurements to experimentally probe efficient plasmon-induced charge transfer in gold nanorods@TiO_2_ core-shell heterostructures (AuNRs@TiO_2_). Specifically, we quantify the contribution of direct charge transfer by measuring the plasmon decay for individual AuNR@TiO_2_ heterostructures (Fig. 1C) and achieve excellent agreement with Persson’s model of CID (*36*). We then compare CID values with total (indirect + direct) electron injection efficiencies by probing free carrier absorption in the conduction band of TiO_2_ using variable visible pump—infrared (IR) and near-infrared (NIR) probe transient absorption spectroscopy of AuNR@TiO_2_ films (Fig. 1D). We find quantitative agreement between the total efficiencies of electrons injected into the conduction band of the semiconductor and electrons extracted from the metal, using a visible probe that follows competing hot electron relaxation inside the metal (Fig. 1E).

## RESULTS

We selected AuNR@TiO_2_ heterostructures, illustrated in the schematics in Fig. 1 (C to E), as our platform to distinguish and quantify the different charge transfer pathways because they have ideal material and geometry properties for plasmon-induced electron transfer, and because this material combination is well known to support interfacial charge transfer (*13*, *17*, *22*). The TiO_2_ has suitable n-type semiconductor criteria to facilitate electron conduction to the surface for photocatalytic applications (*41*), while its wide bandgap (~3.2 eV) excludes the possibility of visible light excitation of electrons from the valance band to the conduction band (*42*). Furthermore, the interfacial Schottky barrier (0.9 to 1.2 eV) formed at the Au-TiO_2_ interface can be easily exceeded by the tunable LSPR of AuNRs, thereby prolonging the lifetimes of the injected hot electrons for enabling redox reactions or promoting photocurrents (*9*, *26*). In addition, using a chemically grown core-shell architecture with its intimate interface and the large active interfacial area has been proposed as a beneficial approach to facilitate charge transfer (*43*, *44*). Last, AuNRs as the core offer superior plasmonic properties compared to commonly studied Au nanospheres. The AuNR’s anisotropic geometry should facilitate direct electron transfer by providing the necessary momentum through strong electric field confinement (*45*–*47*), while the AuNR LSPR can be tuned away from Au interband transitions that cause additional damping (*48*). The AuNR@TiO_2_ heterostructures studied here had an average core dimension of 26 ± 2 nm × 49 ± 3 nm and an average TiO_2_ shell thickness of 28 ± 2 nm and were fabricated by first colloidal synthesis of the AuNR core and then solution phase overgrowth of the TiO_2_ layer. This AuNR size provided sufficient scattering for quantitative single-particle dark-field scattering spectroscopy with a TiO_2_ shifted resonance that is lower energy than the Au interband threshold to minimize bulk damping contributions to the plasmon linewidth while remaining within the spectral window of our instruments. The chosen shell thickness avoided plasmon coupling between adjacent nanoparticles for the AuNR@TiO_2_ films used in ensemble studies since coupling strength scales with interparticle distance cubed (*49*).

### Single-particle scattering spectroscopy reveals plasmon-induced electron transfer

**Fig. 2.**
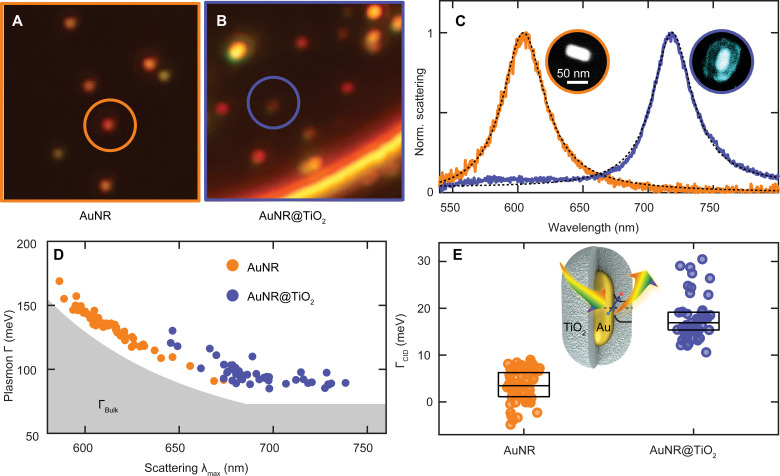
Single-particle dark-field scattering spectroscopy to determine CID. (**A**) Dark-field scattering image of single AuNRs. (**B**) Dark-field scattering image of single AuNRs@TiO_2_. (**C**) Normalized correlated single-particle scattering spectra of a representative single AuNR (orange) and AuNR@TiO_2_ (blue) with core sizes of 25 × 53 ± 2 nm and 25 × 55 ± 2 nm and their Lorentzian fits (black dashed lines). Inset: Color-coded correlated electron micrographs. (**D**) Plasmon linewidth (Γ) as a function of scattering maximum (λ_max_) calculated from Lorentzian fits for single AuNRs (orange) and AuNRs@TiO_2_ (blue). The gray shaded area represents the bulk damping intrinsic to Au (Γ_Bulk_). Error estimates from the fits for λ_max_ and Γ are roughly the size of the data points. (**E**) Distributions of Γ_CID_ values for AuNRs (orange) and AuNRs@TiO_2_ (blue) obtained from the difference between measured Γ and size-dependent bulk and radiation damping for every single particle based on correlated electron microscopy. Inset: Schematic illustration of the experimental design for investigating CID in AuNR@TiO_2_ heterostructures. Data points of individual AuNRs and AuNRs@TiO_2_ are distributed along the *x* axis for visualization. The box plots represent the interquartile range and the median of the distributions. The AuNRs@TiO_2_ exhibit significant Γ_CID_ (19 ± 7 meV) compared to the bare AuNRs (3 ± 4 meV); *t*(108) = 14.8, *P* < 0.0001.

We isolated individual nanostructures in correlated optical and electron microscopy using indexed quartz substrates, following previously established procedures (*50*). Figure 2 (A and B) illustrates dark-field scattering images of single AuNRs and single AuNRs@TiO_2_, respectively. After identifying the positions of the individual particles based on their integrated intensity, the scattered light was directed toward a spectrograph equipped with a camera to obtain their homogeneous plasmon linewidths (*50*–*52*). Representative spectra for a single AuNR and AuNR@TiO_2_ with similar sizes are compared in Fig. 2C, while their corresponding dimensions were obtained by scanning electron microscopy (Fig. 2C, insets). The LSPRs are red-shifted for the AuNRs@TiO_2_ compared to AuNRs because of the higher refractive index of the TiO_2_ shell compared to air (*53*). High-resolution transmission electron microscopy reveals that the initially amorphous TiO_2_ is converted to anatase TiO_2_ upon thermal annealing at 500°C for 1 hour (fig. S1).

We quantified the direct charge transfer pathway for all individual AuNRs@TiO_2_ by accounting for CID contributions to the overall homogeneous plasmon linewidth, determined from Lorentzian fitting (Fig. 2C). The total plasmon damping (Γ) for each nanoparticle, equal to the resonance plasmon linewidth, is depicted in Fig. 2D for 59 AuNRs and 51 AuNRs@TiO_2_ as a function of their respective LSPR wavelength. The direct charge transfer pathway provides an additional decay channel for plasmon damping through CID, hence affecting the total scattering linewidth (*36*, *53*, *54*), while indirect charge transfer remains invisible in the single-particle spectrum because it occurs after plasmon decay. The contribution of only direct charge transfer through CID to the broadening of the plasmon linewidth (Γ_CID_) was isolated and quantified according to a phenomenological model that adds up all possible plasmon damping channels, i.e., Γ = Γ_Bulk_ + Γ_rad_ + Γ_CID_ (*54*–*56*). These decay channels contributing to the total linewidth include resonance-dependent bulk Au damping, Γ_Bulk_, indicated by the gray area in Fig. 2D, and size-dependent radiation damping (Γ_rad_). Γ_Bulk_ was calculated from the energy-dependent bulk dielectric function, while Γ_rad_ was accounted for by the nanoparticle volume (see the Supplementary Materials for more details) (*51*, *55*, *57*). Γ_CID_ for the AuNRs and AuNRs@TiO_2_ was then simply obtained by subtracting Γ_Bulk_ and Γ_rad_ from the measured LSPR linewidths.

The efficiency of direct plasmon-induced charge transfer through the CID channel is estimated to be 19 ± 4% according to η_Direct_ = Γ_CID_/Γ for our AuNR@TiO_2_ system. The calculated values of Γ_CID_ are shown in Fig. 2E, grouped separately for AuNRs and AuNRs@TiO_2_. Γ_CID_ calculated for AuNRs (Fig. 2E, orange) has a negligible but nonzero value of 3 ± 4 meV, which is likely due to the neglected electron-surface scattering. Therefore, Γ_CID_ of the bare AuNRs serves as a reference for no charge transfer. Γ_CID_ of the AuNRs@TiO_2_ has a mean value of 19 ± 7 meV, indicating that CID causes substantial plasmon damping through the direct charge transfer mechanism. We can further assign a corresponding transfer time of 70 ± 40 fs, assuming that dephasing is dominated by energy instead of phase relaxation (*48*, *58*). However, it must be noted that charge transfer is only one of several possible explanations for CID (*54*). Thus, a multimodal approach is needed to corroborate the direct charge transfer efficiency and to quantify its contribution to the total charge transfer efficiency.

### IR transient spectroscopy quantifies total efficiency of injected electrons into TiO_2_

**Fig. 3.**
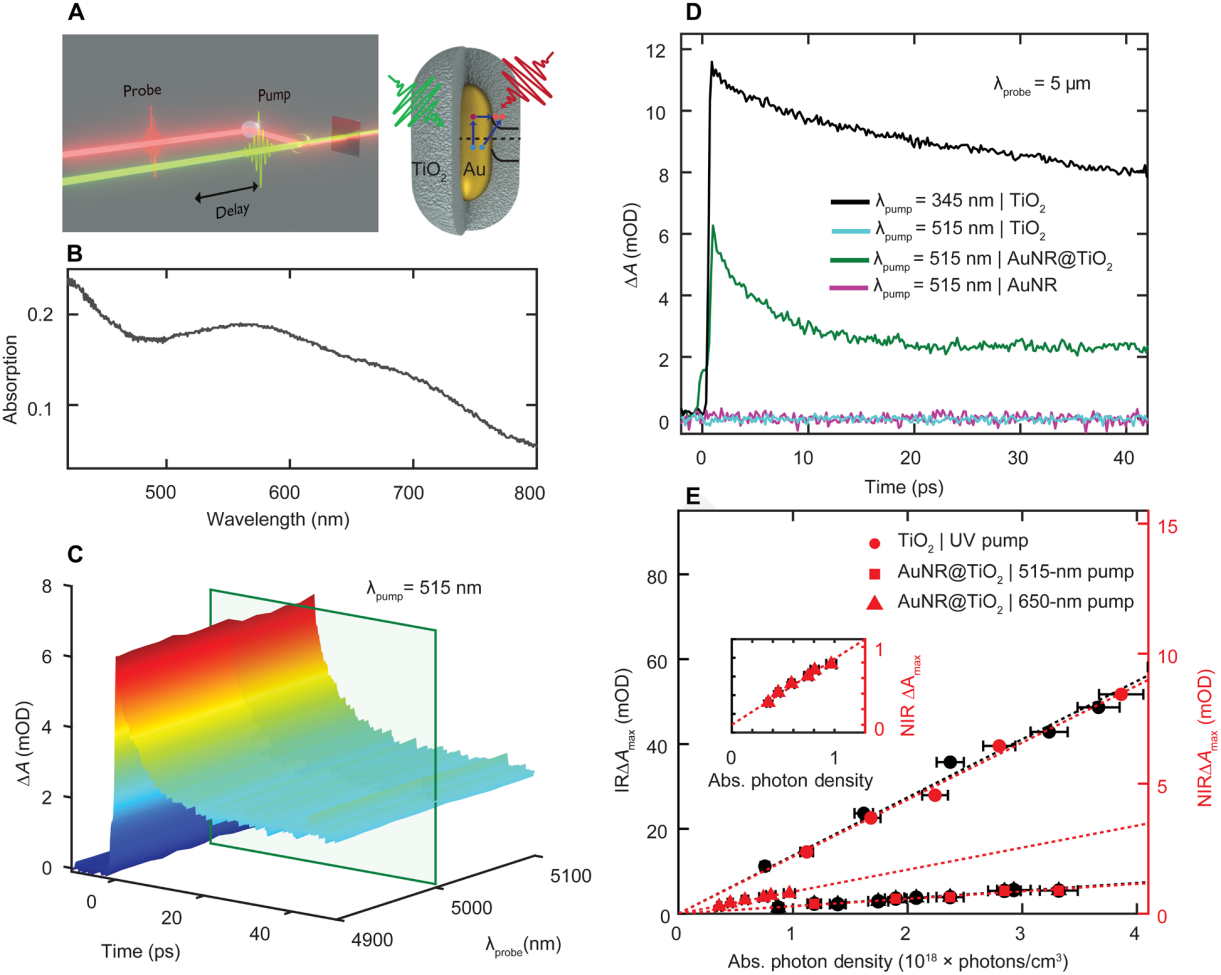
IR and NIR transient absorption spectroscopy of injected electrons in the conduction band of TiO_2_. (**A**) Experimental setup and cartoon illustration of visible pump and NIR or IR probe transient absorption spectroscopy to interrogate the dynamics of injected electrons through direct and indirect charge transfer into the TiO_2_ conduction band for AuNR@TiO_2_ heterostructures. (**B**) Steady-state absorption spectrum of a AuNR@TiO_2_ film. (**C**) Ultrafast IR transient absorption spectra of AuNR@TiO_2_ heterostructures probed at 4.9 to 5.1 μm following 515-nm excitation in units of mili optical density (mOD). The green box indicates the probe wavelength of 5 μm used for analyzing the dynamics shown in (D). (**D**) Transient absorption dynamics at 5 μm for TiO_2_ with 345-nm (black) and 515-nm excitation (cyan), AuNRs@TiO_2_ with 515-nm excitation(green), and AuNRs with 515-nm excitation (pink). (**E**) Experimentally derived values of Δ*A*_max_ are shown as a function of absorbed (abs.) photon density using pump fluence–dependent experiments for the TiO_2_ film with 345-nm pump and 5-μm probe (black circles) and for AuNRs@TiO_2_ with 515-nm pump and 5-μm probe (black squares). Similar experiments with a 1.15-μm probe are shown for TiO_2_ excited with 266-nm pump (red circles) and for AuNRs@TiO_2_ with 515-nm pump (red squares) and 650-nm pump (red triangles and inset) as a function of absorbed photon density. Table S1 lists the fitted slopes.

The injection of electrons into the TiO_2_ shell was examined by IR transient absorption pump-probe spectroscopy (Fig. 3A). For these measurements, films were prepared, and the frequency resolved absorption of the ensemble AuNR@TiO_2_ sample was determined on the basis of its reflection and transmission spectra measured using an integrating sphere (Fig. 3B and fig. S2). The absorption spectra of AuNR@TiO_2_ (Fig. 3B) and AuNR (fig. S2) films displayed a broad LSPR centered at 700 and 670 nm, respectively, with a transverse LSPR at 550 nm for both samples. The LSPRs are wider in these film samples compared to those in the single-particle spectra due to inhomogeneous broadening associated with ensemble measurements (*54*). Minor aggregation especially for the bare AuNRs can further contribute to the inhomogeneous LSPR linewidth, although the TiO_2_ shell kept the AuNR cores at least ~60 nm apart, preventing strong near-field coupling that could influence hot carrier generation (*49*, *59*). The AuNR@TiO_2_ sample exhibited a featureless IR transient absorption signal around 5 μm with an amplitude that is proportional to the electron density in the conduction band of the semiconductor (*16*, *60*). A characteristic time-resolved spectrum of the plasmon-induced electron injection after photoexcitation of the AuNR@TiO_2_ heterostructures with a 515-nm pump pulse is depicted in Fig. 3C. A pump wavelength of 515 nm (*h*ν = 2.4 eV) produced electrons with sufficient energy to overcome the Schottky barrier between the Au and TiO_2_ interface (*61*, *62*). The transient IR signal follows a multiexponential decay, resulting eventually in charge recombination on a time scale faster than the laser repetition rate so that overall charge neutrality was maintained (*16*).

Photoexcitation of the AuNRs@TiO_2_ induced ultrafast electron injection into the TiO_2_ shell. Time-resolved spectral slices at 5 μm are depicted in Fig. 3D for the AuNR@TiO_2_ sample as well as AuNR and TiO_2_ controls. Following 515-nm excitation, the transient absorption signal of the AuNRs@TiO_2_ displays a rise (Fig. 3D, green line) that is within the ~200-fs temporal resolution of our experimental setup (fig. S3), consistent with previous reports (*13*, *17*). Although we can therefore conclude that charge injection from the metal into the conduction band of the TiO_2_ occurs, the underlying electron transfer pathways (direct or indirect charge transfer) cannot be distinguished on this timescale. Exciting the TiO_2_ film above its bandgap at 345 nm also shows a steep, instrument-limited rise due to free carrier absorption within the conduction band of the TiO_2_ followed by a similar decay (Fig. 3D, black line). However, when exciting below the TiO_2_ bandgap at 515 nm, no signal is observed (Fig. 3D, cyan line), confirming that the 515-nm excitation is below the bandgap of TiO_2_. To further ensure that the measured transient absorption signal for 515 nm excitation of AuNRs@TiO_2_ arises from the electrons within the conduction band of the TiO_2_ and does not originate from the AuNRs, we investigated the transient absorption of bare AuNRs. As shown in Fig. 3D (pink line), no measurable transient absorption signal was detected for 515-nm excitation of the bare AuNRs on a sapphire (Al_2_O_3_) substrate. The interfacial Schottky barrier for Au and Al_2_O_3_ is approximately ~4 eV and hence larger than the 515-nm photon energy (*63*). Last, the multiexponential decay of the IR transient absorption signal with lifetimes of ~1 and ~100 ps is due to charge recombination, likely mediated by defect states, and agrees with previous reports (*16*, *19*).

Electron injection efficiencies were quantified by accounting for the ratio of the number of electrons injected into the semiconductor to the total number of excited electrons within the metal. The total number of excited electrons within the metal is proportional to the number of absorbed photons, which can be determined from the excitation photon energies, pump fluences, and optical densities (*13*). Above-bandgap (345 nm) fluence-dependent pumping of a TiO_2_ film was used as a reference (fig. S4), assuming that each absorbed pump photon excited one electron into the conduction band of TiO_2_ and the 5-μm transient absorption signal intensity at zero pump-probe delay times, Δ*A*_max_, scales linearly with the maximum number of free conduction band carriers. Figure 3E (black circles) confirms this linearity between Δ*A*_max_ and the absorbed photon density (see the Supplementary Materials for more details). By fitting the data to a linear regression, a slope *m*_TiO_2__ = 12.5 ± 0.6 mili optical density (mOD) × cm^3^ per photon × 10^−18^ was determined, while for 515-nm excitation of the AuNRs@TiO_2_ we obtained a smaller slope *m*_AuNR@TiO_2__ = 1.9 ± 0.1 mOD × cm^3^ per photon × 10^−18^ (Fig. 3E, black squares), consistent with a less than 100% charge injection efficiency for the AuNR@TiO_2_ heterostructures. The total electron injection efficiency for the AuNRs@TiO_2_ with 515-nm excitation was then calculated according to η_Indirect + Direct_ = *m*_AuNR@TiO_2__/*m*_TiO_2__, resulting in a value of 15 ± 6%. It is important to note that this efficiency is the absorbed photon quantum efficiency and not an incident photon quantum efficiency, which is lower. Furthermore, this charge transfer efficiency is quite high, indicating that, in addition to the pathways illustrated in Fig. 1, a plasmon-independent direct absorption pathway could also exist between gold and TiO_2_.

The importance of LSPR excitation on electron injection efficiencies was identified by tuning the excitation wavelength in the visible region and probing the dynamics of AuNRs@TiO_2_ at a NIR wavelength of 1.15 μm. Although free carrier absorption decreases with decreasing wavelength, NIR probing has been reported to still be sensitive only to electrons within the conduction band of TiO_2_ (fig. S5) (*60*) and allowed us to use a separate ultrafast laser system with broad pump wavelength tunability. Repeating the above-described series of pump fluence–dependent transient absorption measurements, we indeed observed a similar injection efficiency of 18 ± 1% when using a 1.15-μm probe for 515-nm excitation of the AuNRs@TiO_2_ and 266-nm excitation of the TiO_2_ (Fig. 3E, red squares). However, when exciting the AuNRs@TiO_2_ close to their LSPR at 650 nm (Fig. 3E, red triangles), an overall electron injection efficiency of η_Indirect + Direct_ = 44 ± 3% was achieved, despite 515-nm photons having a larger energy. We assume and further prove below that this substantially increased electron injection efficiency by exciting at the LSPR at 650 nm is caused by the additional contribution from the direct interfacial charge transfer pathway that is only possible with LSPR excitation. Since we are looking at internal efficiencies, thus accounting for the absorptivity at different pump energies, we can exclude simple absorption enhancement at the LSPR.

### AuNR electron-phonon relaxation times augmented by electron transfer into TiO_2_

**Fig. 4.**
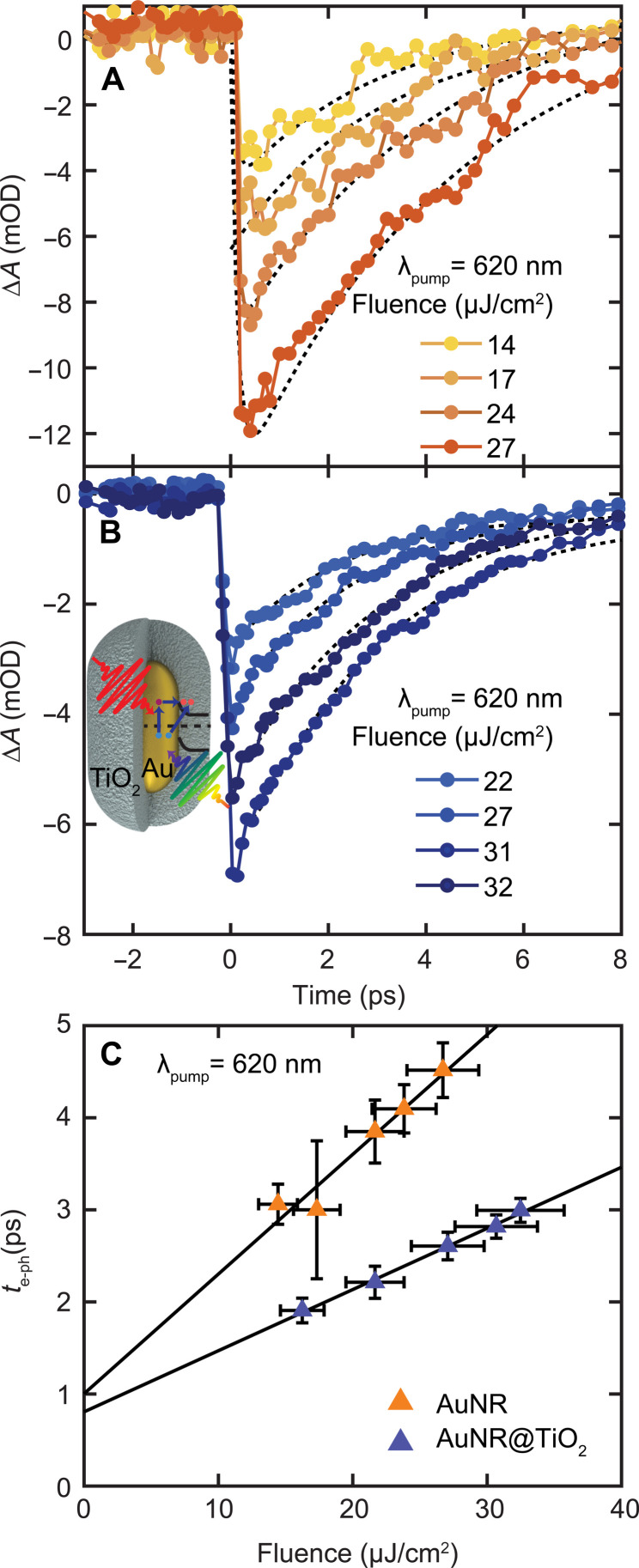
Influence of electron injection on the dynamics of hot carriers inside the AuNR core. (**A**) Fluence-dependent bleach recovery dynamics for AuNRs with 620-nm excitation and monitored at 685 nm as a function of pump fluence, as indicated in the legend. (**B**) Same as (A) for AuNRs@TiO_2_ and probed at 715 nm, following changes in the dynamics of hot carriers inside the AuNR core after electron injection through direct and indirect charge transfer from the AuNR core to the TiO_2_ shell, as indicated by the schematic illustration in the inset. (**C**) Electron-phonon relaxation times τ_e–ph_, extracted from fitting the plasmon recovery dynamics to the two-temperature model, as a function of incident pump fluence for AuNRs (orange) and AuNRs@TiO_2_ (blue) for 620-nm excitation. Table S1 lists the fitted slopes.

The electron dynamics of Au nanoparticles have been studied extensively (*6*, *7*, *64*–*67*), and the ultrafast carrier dynamics inside the AuNR core can be well described using the two-temperature model (*68*, *69*). Photoexcitation causes a bleach at the LSPR with recovery dynamics that are governed initially by electron-electron scattering as the excited electrons thermalize to form a hot electron distribution with an elevated electron temperature described by Fermi-Dirac statistics (*70*). The hot electrons simultaneously equilibrate with the lattice through electron-phonon coupling on a ~1-ps timescale, forming a lower electron temperature equal to the now raised lattice temperature and yielding the main LSPR bleach recovery signal in the visible transient absorption spectrum (*67*, *70*). The extracted bleach recovery dynamics, however, depend linearly on the initial thermalized electronic temperature *T*_e_ (for *T*_e_ < ~3000 K in Au) (*67*) and hence the total photon energy absorbed because the electronic heat capacity is temperature dependent (*6*, *69*, *71*). Therefore, to investigate the effects of electron injection from the AuNR core on the dynamics of the remaining electrons inside the metal, we studied the bleach recovery dynamics of bare AuNRs as a reference (Fig. 4A) and compared it to the behavior of AuNRs@TiO_2_ (Fig. 4B) as a function of pump fluence. Figure 4 summarizes the results using 620-nm excitation with probe wavelengths of 685 and 715 nm for AuNRs and AuNRs@TiO_2_, respectively, corresponding to their respective LSPR maxima. Probing close to the LSPR maxima provided the highest sensitivity but required us to slightly detune the pump for on-resonance excitation within the heterogeneously broadened absorption to avoid interference from scattered pump light. The bleach recovery dynamics were modeled using the two-temperature model, and the extracted electron-phonon relaxation times τ_e–ph_ could be described by a linear dependence on pump fluence for the excitation regime used (Fig. 4C, blue and orange triangles for the AuNR@TiO_2_ and AuNR samples, respectively). In general, the measured relaxation times are consistent with previous observations for Au nanoparticles (*6*, *13*, *71*–*73*). However, the values for the heterostructures were found to be consistently lower than the control for each pump fluence.

Ultrafast electron injection from the AuNR core to the TiO_2_ shell substantially alters the carrier dynamics inside the AuNR core, as evident from the difference in slopes of the pump fluence–dependent measurements (Fig. 4C). The efficient injection of an electron into the TiO_2_, established by IR and NIR transient absorption spectroscopy, reduces the initial electronic temperature, causing the electron bath to thermalize to a lower temperature, resulting in shorter measured bleach recovery dynamics for a given pump fluence. The slope is reduced for the AuNRs@TiO_2_ compared to the AuNRs (Fig. 4C), indicating a lower electronic temperature, which we attribute to the ultrafast electron injection across the Schottky barrier in the AuNRs@TiO_2_. The relative ratio of the slopes can be used to estimate the fraction of electrons collected by the TiO_2_ shell upon excitation of the AuNRs (see the Supplementary Materials for more details) (*13*). The reduction in the slope of the fluence-dependent τ_e–ph_ for the AuNRs@TiO_2_ upon 620-nm excitation is 49 ± 3%. This value is in very good agreement with the transfer efficiency of 44 ± 3% determined by NIR transient absorption spectroscopy that probes the complementary free electron absorption in the conduction band of TiO_2_. Charge injection efficiencies probed in the visible for 400- and 515-nm excitation following the same analysis (fig. S6) resulted in overall electron injection efficiencies of 17 ± 5% and 22 ± 2%, respectively. These values are again in excellent agreement with the injection efficiencies measured using NIR transient absorption spectroscopy, further corroborating our approach and confirming that other enhanced absorption mechanisms such as sub-bandgap or direct absorption play no or only a minor role.

### Plasmon-induced direct charge transfer greatly increases electron transfer efficiency

**Fig. 5.**
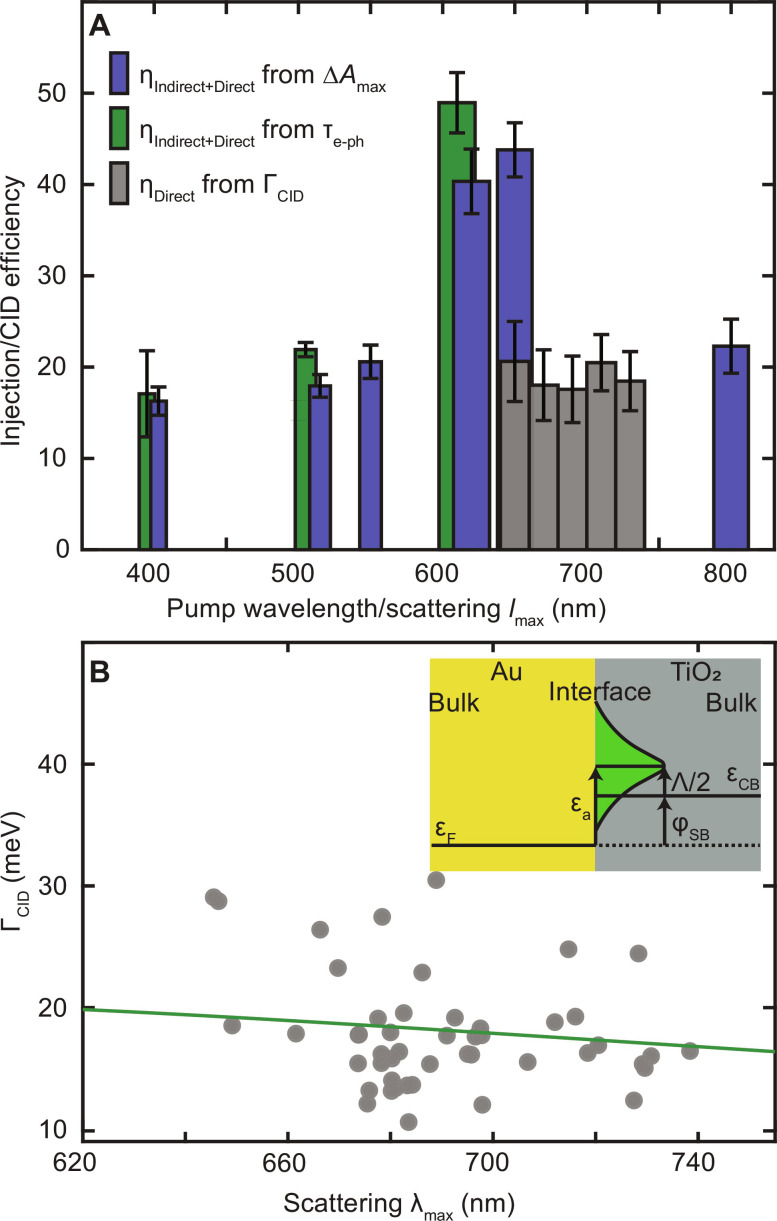
Plasmon excitation boosts interfacial charge transfer in AuNR@TiO_2_ heterostructures. (**A**) Efficiencies of charge injection at different excitation wavelengths obtained from NIR transient absorption spectroscopy excited at 1.15 μm (blue bars), determine the sum of direct and indirect charge transfer pathways by monitoring free carrier absorption in the TiO_2_ conduction band. Efficiencies of total (direct + indirect) charge transfer, (green bars) calculated from the changes in slopes of pump fluence–dependent electron-phonon relaxation times τ_e–ph_ using a visible probe at the LSPR maximum. The widths of the blue and green bars reflect the spectral width of the excitation, and the error bars are the fit uncertainties. The spectrally resolved efficiencies of the isolated direct charge transfer pathway (gray bars) obtained through quantifying CID as a function of single-particle resonance maxima λ_max_. The CID efficiencies are binned with a width of 20 nm, and the error bars are the SD of the single-particle data. (**B**) CID dependence on scattering λ_max_. Gray points are CID values from Fig. 2E, while the green line is the predicted wavelength dependence of CID according to the Persson model. Inset: Illustration of adsorbate-induced resonance state formation at the Au-TiO_2_ interface according to the Persson model.

The electron injection efficiencies for AuNR@TiO_2_ heterostructures greatly increase when exciting near the LSPR, although we have fully accounted for differences in absorption. Figure 5 summarizes the calculated efficiencies as a function of excitation wavelength determined from all three experimental platforms described above (Fig. 1). The electron injection efficiencies obtained from NIR transient absorption spectroscopy cover pump wavelengths that coincide with interband transitions only (400 nm), mainly interband transitions and a weak transverse LSPR (515 and 550 nm), the strong longitudinal LSPR (620 and 650 nm), and intraband transitions (800 nm), yielding values of 17 ± 2%, 18 ± 1%, 21 ± 2%, 40 ± 4%, 44 ± 3%, and 22 ± 3%, respectively (Fig. 5A, blue bars). The electron injection efficiencies for near LSPR excitation (620 and 650 nm) exceed those when pumping at 400, 515, 550, and 800 nm. We also observed that exciting near the weaker transverse LSPR results in a higher injection efficiency than for pure interband excitation at 400 nm. The complementary analysis following the decrease in hot electrons inside the AuNR core through plasmon bleach dynamics is given by the green bars in Fig. 5A and confirms that efficiencies are enhanced by LSPR excitation. This excitation energy dependence is inconsistent with Fowler’s equation for predicting the electron transfer through the indirect electron transfer pathway, in which the injection efficiency is expected to increase at shorter excitation wavelengths due to the excess energy of the hot electron (*15*). We note that Fowler’s model cannot fully describe the indirect electron transfer pathway as it does not consider the impact of interband excitation on the barrier height nor does it account for Auger scattering efficiently producing hot sp-band electrons from initially excited d-band holes in <100 fs (*55*, *74*).

Considering now the single-particle homogeneous plasmon damping (Fig. 2C) assigned to CID that is only sensitive to the direct charge transfer pathway and can only occur at the LSPR, we conclude that the excitation energy-dependent total charge transfer efficiency extracted from ultrafast spectroscopy originates from the additional contribution of direct plasmon decay into interfacial charge separated states. CID efficiencies are plotted as a function of LSPR wavelengths, binned in 20-nm intervals (Fig. 5A, gray bars). Comparing η_Indirect + Direct_ as derived from transient absorption spectroscopy and η_Direct_ as derived from CID indicates that the direct charge transfer pathway contributes about 40 to 50% to the overall efficiency of injected electrons from the AuNR core to the TiO_2_ shell at the LSPR. Thus, the direct charge transfer pathway is a major contributor to the total electrons injected and the underlying reason why charge injection is increased when exciting on resonance, while the indirect charge transfer pathway happens at all wavelengths, as evident from the almost constant value of ~20% regardless of whether interband or intraband transitions are excited. While interband transitions initially generate hot d-band holes and cool sp-band electrons (*75*, *76*), the d-band holes quickly scatter to produce hot sp-band electrons (*62*, *74*). Thus, our results of comparable injections efficiencies at 400 and 800 nm indeed imply that similar distributions of hot electrons are produced within roughly the duration of the pump pulse by exciting interband (400 nm) versus pure intraband (800 nm) transitions.

### Interface-induced resonance states mediate plasmon-induced direct charge transfer

Thus far, we have implied that the measured CID corresponds to direct charge transfer, although CID is a rather general description of an additional plasmon damping process that could also be due to plasmon image dipole scattering and resonance energy transfer (*54*). Energy transfer can be ruled out based on the much larger TiO_2_ bandgap than the plasmon energy (*77*). Furthermore, charge injection was confirmed by free carrier absorption in the IR and NIR (Fig. 3) and by the quantitative agreement between CID efficiencies and the increase in total charge injection efficiency at the LSPR (Fig. 5A). Nevertheless, we also applied a theoretical model proposed by Persson (*36*), to firmly establish the mechanism of CID for the AuNR@TiO_2_ heterostructures. This model provides a quantitative estimate of Γ_CID_ under the assumption that the broadening arises from charge transfer to adsorbate-induced resonance states (see the Supplementary Materials for more details). The computed Γ_CID_ is 21 meV, which is in excellent agreement with the measured range of 19 ± 7 meV. In addition, the model predicts a weak LSPR energy dependence for CID that matches well with the experimental data (Fig. 5E). Thus, we have established that direct charge transfer across the interface is responsible for the experimentally observed LSPR broadening.

In these calculations, we assumed that there is one resonance state per surface Au atom, i.e., that the resonance states of interest are inherent to the interface and not associated with isolated interfacial defect sites at low concentration. To test the hypothesis that the broadening may instead arise from isolated defects, we computed Γ_CID_ for hypothetical systems with a range of defect state energies (0 to 500 meV below the TiO_2_ conduction band) and surface defect concentrations (0.01 to 0.1 defects per surface Au atom). Computed Γ_CID_ values span the range of 0.2 to 2.8 meV (see table S2), implying that Γ_CID_ scales linearly with the surface concentration of resonance states. Given the much poorer agreement with experiment for these conditions, we conclude that the broadening must arise from resonance states inherent to the interface rather than from a smaller number of isolated defect states.

## DISCUSSION

In summary, we used three different imaging and spectroscopy strategies and a theoretical model to obtain electron injection efficiencies for AuNR@TiO_2_ core-shell heterostructures with average dimensions of 26 nm × 49 nm. Plasmon enhanced electron injection efficiencies of 44 ± 3% were determined using NIR transient absorption spectroscopy. The electron injection efficiencies were significantly reduced when exciting the AuNR@TiO_2_ heterostructures off-resonance (17 ± 2%), emphasizing the role of plasmon excitation, *t* (4) = 12.97, *P* = 0.0002. We corroborated these results further by separately investigating the effect of electron injection on the electronic temperature inside the AuNR cores. By single-particle imaging and spectroscopy, we obtained the homogeneous plasmon linewidth broadening, from which we determined an approximate 20% efficiency for the direct electron injection pathway by analysis of plasmon damping. Therefore, about half of the total injected electrons are from the plasmon decaying directly into interfacial charge-separated states through a direct electron transfer mechanism. It is important to emphasize that, since cross sections were specifically accounted for in the reported efficiencies, enhancement at the plasmon resonance is due to the direct electron transfer pathway becoming available and not because of stronger absorption by the plasmon. The plasmon-induced direct charge transfer mechanism was modeled using Persson’s theory of adsorbate-induced resonance states allowing for predictive direct charge transfer efficiencies for various plasmonic nanoparticles and semiconductors. Critically, Persson’s theory identifies the energy of the adsorbate state, the LSPR energy, and the nanoparticle size as important parameters for future optimization of interfacial charge injection (fig. S9). Our observations provide conclusive evidence for the direct carrier injection pathway, advancing mechanistic understanding of the charge transfer in metal-semiconductor heterostructures. The knowledge gained here will allow for a more rational design of devices based on specially designed plasmonic nanoparticles by circumventing the metal intrinsic energy loss channel of nonradiative relaxation through electron-phonon coupling that leads to unwanted heating.

## MATERIALS AND METHODS

### Experimental design

AuNR@TiO_2_ heterostructures have ideal material and geometry properties for plasmon-induced electron transfer. The interfacial Schottky barrier formed at the Au-TiO_2_ interface is easily exceeded by the tunable LSPR of AuNRs. The AuNR’s anisotropic geometry should furthermore facilitate direct electron transfer by providing the necessary momentum through strong electric field confinement, while the AuNR LSPR can be tuned away from Au interband transitions that cause additional damping. By comparing the frequency-resolved, single-particle scattering spectra of bare AuNRs to AuNRs@TiO_2_, we can identify the direct charge transfer efficiency. Then, by time-resolving the dynamics of the AuNRs@TiO_2_ in comparison to AuNR and TiO_2_ samples, we can quantify the total charge transfer at different pump wavelengths. Last, the combination of frequency- and time-resolved methods allows for the total charge transfer efficiency to be decomposed into contributions from the direct and indirect charge transfer pathways.

### Synthesis of AuNRs and AuNR@TiO_2_ core-shell heterostructures

The AuNR sample was synthesized using a seed-mediated growth method according to (*78*). The AuNR size distribution and the synthesis protocols are described in a previous publication (*47*). The average AuNR dimensions were 29 ± 2 nm × 63 ± 3 nm. The core-shell AuNR@TiO_2_ nanoparticles were prepared in three steps: ligand exchange, growth of an amorphous TiO_2_ shell, and thermal annealing. First, ligand exchange was carried out according to (*79*). A total of 0.05 ml of AuNR solution was mixed with 1 ml of 0.1 M SDS (ACS reagent, ≥99.0%; Sigma-Aldrich) solution and stirred for 30 min. The unreacted SDS molecules were separated using centrifugation, and the AuNRs were redispersed in 0.5 ml of H_2_O. Second, Ti^3+^ precursor solution was prepared using 0.1 ml of 12% TiCl_3_ in 4 ml H_2_O. A total of 0.5 ml of 1 M NaHCO_3_ was added to the TiCl_3_ solution dropwise under stirring until the color of the solution turned dark blue. The pH of the solution at this condition was ~2.5. Then, 0.5 ml of ligand-exchanged AuNR solution was added to the dark blue solution and stirred for 1 min at room temperature. The AuNR@TiO_2_ solution was washed with pure water by centrifugation, and most of the unreacted Ti^3+^ precursor was removed in this step. The final AuNR@TiO_2_ nanoparticles were dispersed in 1 ml of pure water. Third, the core-shell nanoparticles were thermally treated to crystallize the TiO_2_ shell by placing samples into a box furnace at a temperature of 500°C for 1 hour.

### Sample preparation for single-particle imaging and spectroscopy

An appropriate concentration of AuNR and AuNR@TiO_2_ samples, found by successive dilution, were spin-coated on indexed SiO_2_ substrates for 60 s at 2500 rpm to achieve single-particle coverage. Substrate indexing was performed to enable correlated single-particle measurements by evaporating 35 nm of Au using a labeled alphanumeric grid shadow mask. All substrates were washed, and O_2_ plasma cleaned for 2 min before spin coating. The AuNR@TiO_2_ samples were thermally treated as described above before measurements.

### Sample preparation for transient absorption spectroscopy

AuNR@TiO_2_ samples were prepared for transient absorption measurements by drop-casting an appropriate concentration of the solution onto Al_2_O_3_ substrates (sapphire wafer, c-plane, 2sp, MTI). The substrates were first sequentially cleaned by sonication with isopropanol, methanol, and water for 15 min each, and then O_2_ plasma cleaned for 2 min before drop-casting. Al_2_O_3_ substrates were chosen because of their transmittance in the mid-IR wavelength region. The TiO_2_ control sample was made by drop-casting a solution of commercial TiO_2_ nanoparticles (718467, Sigma-Aldrich) with an average diameter of 21 nm. The AuNR control sample was prepared by drop-casting as well, using the starting AuNR solution. The AuNR@TiO_2_ and TiO_2_ samples were thermally treated as described above.

### Single-particle dark-field imaging and spectroscopy

Single-particle imaging and spectroscopy by dark-field scattering was performed according to previously published studies using an inverted microscope (Axio Observer.D1m, Zeiss) (*47*, *55*). Briefly, an incandescent halogen lamp (HAL 100, Zeiss) was used as excitation light and was focused on the sample by a dark-field oil immersion condenser. The scattered light from the sample was collected with a 50× air spaced objective with a numerical aperture of 0.8 (EC Epiplan-Neofluar, Zeiss). A confocal geometry was achieved by spatially filtering the light through a 50-μm pinhole (Thorlabs) at the first image plane outside the microscope. The sample was positioned using a piezo scanning stage (P-517.3CL, Physik Instrumente). The collected scattered light was first guided to an avalanche photodiode (SPCM-AQRH-15, PerkinElmer) for imaging and acquiring the particle positions and then redirected to a spectrometer (SR193i-A, Shamrock) connected to a charge-coupled device camera (iDus 420 BEX2-DD, Andor) for obtaining spectra. The spectra were corrected by the incandescent light spectrum. A total of 59 AuNR@TiO_2_ and 51 AuNR single particles were measured.

### Transient absorption spectroscopy

The detection of electron injection into the TiO_2_ conduction band was accomplished using IR transient absorption spectroscopy. Briefly, the 1030-nm output of a Yb:KGW regenerative amplifier (Carbide, Light Conversion) was used to pump an optical parametric amplifier (OPA; Orpheus Mid-IR, Light Conversion) that generated signal and idler beams, which were mixed noncollinearly by difference frequency generation in GaSe (Lyra, Light Conversion) to create a 5-μm probe beam with a 250-nm spectral bandwidth. The pump beam was generated from residual of the 1030 nm fundamental accessed from a port of the OPA. Specifically, a 515-nm pump was obtained using second-harmonic generation (SHG) in a β barium borate (BBO) crystal. The 345-nm ultraviolet (UV) pump was generated by third-harmonic generation, wherein the 515-nm SHG signal was mixed with the 1030 nm fundamental to yield a 345-nm beam by sum-frequency generation in a BBO crystal. The 100-kHz output of the Yb:KGW amplifier was reduced to a 1-kHz repetition rate. The pump-probe delay was controlled using a motorized translation stage. A chopper operating at 500 Hz was placed in the pump path to collect pump-on and pump-off spectra. The pump power was controlled using a variable neutral density filter. The pump and probe beams were spatially and temporally overlapped at the focus of a 90° off-axis parabolic mirror. The pump fluence at the sample ranged from 10 to 60 μJ/cm^2^ for 515 nm and from 10 to 40 μJ/cm^2^ for 345 nm. The error bars for the pump fluence were calculated by propagating uncertainties arising from measurements of the laser power and beam area. The mid-IR signal was heterodyne-detected on a HgCdTe array detector (Infrared Associates). Digital to analog conversion was performed using high repetition rate detection electronics (Jackhammer, Phasetech Spectroscopy Inc.).

NIR and visible transient absorption spectroscopy experiments were carried out using a commercial femtosecond transient absorption spectrometer (Helios, Ultrafast Systems). The fundamental 800-nm output of a Ti:sapphire amplifier (Astrella, Coherent) with a repetition rate of 1 kHz was split to drive an OPA (Coherent) for tunable pump pulses in the visible. Pump pulses at 400 and 266 nm were generated from a second- and third-harmonic generator (Ultrafast Systems). Another part of the fundamental output was focused into a CaF_2_ crystal for white-light continuum probe pulses in the NIR and visible spectral regions. The probe wavelength ranged from 450 to 750 nm for the visible white-light continuum and from 850 to 1300 nm for the NIR white-light continuum. The pump pulses were passed through a mechanical chopper to yield a 500-Hz modulation and then focused on the sample. Before reaching the sample, the probe was split into probe and reference beams with equal intensities. The probe and reference beams were detected using a transient absorption spectrometer. To avoid irreversible sample damage, the pump fluence was maintained below 400 μJ/cm^2^. Extinction spectra collected before and after each measurement ensured that the samples were not damaged. Three scans covering 50 ps were collected and averaged for each measurement, with no sample degradation detected from one scan to the next.

### Scanning electron microscopy

Correlated scanning electron microscopy images of all individual 59 AuNRs and 51 AuNRs@TiO_2_ nanoparticles were obtained after the completion of the scattering measurements to determine the dimensions of the nanostructures. Imaging was carried out on a FEI Quanta 400 ESEM FEG in low-vacuum mode operated at a voltage of 30 kV.

### UV-visible absorption spectroscopy

Absorption spectra were determined by measuring the total transmission and reflection of each sample using a spectrometer (J-1500, Jasco) equipped with an integrating sphere. All spectra were recorded under ambient conditions and from the samples used in the transient absorption measurements.

### Calculation of the TiO_2_ contributions to CID

The influence of the matrix environment on the optical absorption and LSPR damping was estimated using the model developed by Persson (*36*). Although this model assumes a spherical particle, we adapted it to the geometry of a prolate cylinder by assuming an identical mean free path, as computed according to (*80*). A more detailed presentation of the theory and the input parameters of the model are provided in the Supplementary Materials.

### Statistical analysis

An independent two-tailed *t* test for the CID values was calculated from the spectra of the 59 individual AuNRs and 51 individual AuNRs@TiO_2_. An independent two-tailed *t* test of the charge transfer efficiency was calculated from the IR transient absorption under 400-nm excitation (*n* = 3) and 650-nm excitation (*n* = 3).

## Supplementary Materials

### This PDF file includes:

Supplementary Text Additional resource for Persson model Figs. S1 to S9 Table S1 and S2 References
