# Supplementary material for: The role of the plasmon in interfacial charge transfer
Source: Sci Adv. 2024 Jul 5;10(27):eadp3353. doi: 10.1126/sciadv.adp3353 (PMC11225779; doi:10.1126/sciadv.adp3353)
Supplement: Supplementary file 1 — Supplementary Text Additional resource for Persson model Figs. S1 to S9 Table S1 and S2 References [file sciadv.adp3353_sm.pdf]

Science Advances
AAAS

Supplementary Materials for

The role of the plasmon in interfacial charge transfer

Behnaz Ostovar *et al.*

Corresponding author: Jennifer A. Dionne, jdionne@stanford.edu; Christy F. Landes, cflandes@illinois.edu; Martin Zanni, zanni@chem.wisc.edu; Benjamin G. Levine, ben.levine@stonybrook.edu; Stephan Link, slink@illinois.edu

Sci. Adv. **10**, eadp3353 (2024)
DOI: 10.1126/sciadv.adp3353

This PDF file includes:

Supplementary Text
Additional resource for Persson model
Figs. S1 to S9
Table S1 and S2
References

Supplementary Text

Charge transfer efficiencies from single-particle spectroscopy

Chemical interface damping (CID) contributions to the measured homogeneous plasmon linewidth Γ were evaluated based on $\Gamma = \Gamma_{Bulk} + \Gamma_{rad} + \Gamma_{CID}$. Γ_{Bulk} was calculated from the energy-dependent bulk dielectric function as described in detail in a previous publication (*55*). Specifically, Γ_{Bulk} was obtained using $\Gamma_{Bulk} = \gamma_b + \epsilon_{IB} E_{res}^3/E_p^2$ where γ_b denotes the Drude damping term, ϵ_{IB} represents the imaginary part of the dielectric function due to interband absorption, E_{res} is the plasmon resonance energy, and E_p is the free electron plasma energy. ϵ_{IB} values were found by subtracting the free electron Drude model from the imaginary part of the dielectric data as determined from Ref. (*57*) with E_p =9.3 eV and γ_b=73 meV according to $\epsilon_{IB} = Im(\epsilon) - (\gamma_b E_P^2)/E_{photon}^3$. The bulk damping contribution to the linewidth is represented by the grey area in Fig. 2C. Γ_{rad} was accounted for according to $\Gamma_{rad} = h\kappa V/\pi$, where h is Planck's constant and a proportionality constant of $\kappa = 3.34 \times 10^{-7}$ fs^{-1} nm^{-3} was used based on previous results for gold nanorods (AuNRs) of similar size on a quartz substrate (*55*). The volume, V, was extracted for each individual AuNR by correlated scanning electron microscopy (*81*). Γ_{CID} was then obtained by subtracting Γ_{Bulk} and Γ_{rad} from the measured linewidth, Γ, for all individual nanoparticles, while the CID efficiency η_{CID}, signifying the direct charge transfer efficiency η_{Direct}, was calculated as the ratio between Γ_{CID} and Γ. We note that plasmon damping due to electron-surface scattering is small enough to be neglected for the dimensions of the AuNRs (26 ± 2 × 49 ± 3 nm) studied here (*55*).

This approach does not consider that Γ_{rad} is expected to also have a resonance energy dependence. Following the method described in Ref. (*82*) we estimate this effect to be on the order of ~5 meV causing a reduction in charge transfer efficiency by 4%, which is on the order of the error for these measurements. In addition, this resonance energy dependence assumes that the nonradiative damping processes are unchanged, i.e. no CID. We therefore decided to neglect this potential additional linewidth contribution, but acknowledge that more detailed studies are needed on systems that show only resonance energy dependent radiation damping compared to those that also support CID.

Charge transfer efficiencies from IR/NIR transient absorption spectroscopy

The total charge transfer efficiencies (direct + indirect pathways) followed by IR/NIR transient absorption of the electrons injected into the TiO_2 conduction band were obtained from fluence-dependent measurement of the signal amplitude for gold nanorod@TiO_2 core-shell heterostructures (AuNRs@TiO_2) compared to direct bandgap excitation for TiO_2 control samples. This analysis assumes that the initial signal at zero pump-probe delay times, ΔA_{max}, is proportional to the free carrier concentration and scales linearly with the absorbed photon density for TiO_2. We accounted for the absorbed photon density based on $n_{ph} = A(\omega_{pump}) \cdot F / \hbar\omega_{pump}$ (*13*). Here ω_{pump} is the pump photon energy and F is the pump fluence. The pump beam diameter was 210 μm at the sample. $A(\omega_{pump})$ is the absorption determined at the pump energy for ech sample according to Fig. S2. In Fig. 3E, the error in absorbed photon densities was calculated by propagating the uncertainties derived from the measurements of pump fluence and beam diameter. The same linearity of ΔA_{max} with absorbed photon density was also observed for the AuNRs@TiO_2, but the slope was smaller (Fig. 3E), consistent with a less than 100% charge injection efficiency. The ratio of slopes between AuNR@TiO_2 and TiO_2 samples yielded the calculated total charge transfer efficiencies.

This analysis, however, neglects that for different excitation wavelengths the electrons injected into the TiO_2 conduction band might have different initial energies, leading to different absorption cross sections, as reported previously for charge injection from ruthenium and rhenium dyes (*83*). While we cannot exclude this possibility, we note that the excellent agreement between the wavelength dependent total charge transfer efficiencies found for probing in the IR/NIR vs. the visible regions (Fig. 5A) suggests that it only plays a minor role for the system studied here. Furthermore, the charge transfer efficiency of 44% at the main plasmon resonance for the AuNRs@TiO_2 agrees well with a value of 20–50% measured for 2–10 nm silver nanospheres on TiO_2 (*19*) and 25–45% measured for 10 nm gold nanospheres (*13*, *17*), when similarly exciting on resonance and using IR transient absorption spectroscopy.

<u>Charge transfer efficiencies from visible transient absorption spectroscopy</u>

The transient bleach recovery dynamics probed at the visible plasmon resonance are proportional to the electronic temperature (*71*, *84*). Interfacial charge transfer lowers the electronic temperature in the AuNR@TiO_2 heterostructures. Taking the ratio of the slopes for the fluence-dependent measurements of the bleach recovery dynamics for AuNRs@TiO_2 compared to AuNRs then yields the charge transfer efficiency (*13*). The bleach recovery dynamics were extracted from the data based on the two-temperature model that has been widely used for metals to model the relaxation dynamics of excited electrons by coupling to a phonon bath through electron-phonon interactions (*2*, *3*, *12–13, 85*). The electron distribution relaxes from an initial nonthermal (NT) to a thermalized (Th) distribution through electron-electron scattering. The temporal evolution of the electron and phonon temperatures upon ultrashort pulse excitation is given by (*68*, *69*): $C_e(T_e)\frac{dT_e}{dt}=-g(T_e-T_l)$ and $C_l\frac{dT_l}{dt}=g(T_e-T_l)$, where, $C_e=\gamma T_e$ is the temperature-dependent electronic heat capacity, which has a linear relationship with the temperature in the low-temperature regime (< ~3000 K for Au) (*67*, *86*). γ is the electron heat capacity constant (66 $Jm^{-3}K^{-2}$ for Au). C_l represents the lattice heat capacity (*68*, *87*). g denotes the electron-phonon coupling constant. T_e and T_l are the electronic and lattice temperatures, respectively. We employed this two-temperature model to quantify the plasmon bleach dynamics expressed as the differential transient transmission $\Delta T/T\ (h\nu,t)$ according to (*40*, *84*): $\Delta T/T\ (h\nu,t) = [\Delta T/T\ (h\nu)]_{NT}\cdot e^{(-t/\tau'_{th})} + [\Delta T/T\ (h\nu)]_{Th}\cdot e^{(-t/\tau_{e-ph})}\cdot\{1-e^{(-t/\tau_{th})}\}$, where $1/\tau'_{th} = 1/\tau_{th} + 1/\tau_{e-ph}$. τ_{th} defines the thermalization time of electrons. The temperature of the thermalized electron distribution decreases due to electron-phonon interactions with a time of τ_{e-ph}. By fitting of the data to this model, we obtained τ_{e-ph} as a function of pump fluence at different excitation wavelengths for the AuNR@TiO_2 heterostructures and AuNR controls.

It needs to be stressed though that the linearity of τ_{e-ph} is with respect to T_e, which in turn depends on the square root of the total energy absorbed from the pump beam determined by the nanoparticle absorption crosse section and the incident fluence (*71*). However, for the range of pump fluences used here, an approximate linear dependence between τ_{e-ph} and absorbed fluence holds, as seen in Figs. 4C and S6. Furthermore, plotting τ_{e-ph} against incident instead of absorbed fluence is justified here because we adjusted the optical densities of the AuNR@TiO_2 and AuNR films to be the same with respect to the contribution from the Au (see Figs. 2G-2I, i.e. difference between 2G and 2H compared to 2I). Because of the broad surface plasmon resonance (SPR) of the films this approach is furthermore justified for the different excitation wavelengths.

Transmission electron microscopy of $AuNRs@TiO_2$

The $AuNR@TiO_2$ samples were prepared for transmission electron microscopy by drop-casting an appropriate concentration of the solution onto 5 nm thick SiN window grids (SN100H-A05Q33A, SiMPore). Samples were imaged after subjecting them to the same thermal annealing conditions as described in the main text, following their deposition on the grids. Transmission electron microscopy was performed using a FEI Titan ETEM operated at a 300 kV acceleration voltage. Images were captured using a Gatan OneView and K3 camera. Selected area electron diffraction was performed using a 10 μm aperture to select < 10 nanorods for a single diffraction pattern. Diffraction simulation analysis was performed using the SingleCrystal software package and reference library. Selected area electron diffraction images were converted into .tif files and imported to SingleCrystal. The Au <111> diffraction peaks were used to calibrate the image dimensions within SingleCrystal. Then the simulated powder diffraction pattern was overlaid for Au and rutile and anatase TiO_2 revealing good agreement for anatase, which has superior charge injection properties (*88*). Fourier transforms were computed using the full frame images in Figs. S1B and S1F within Gatan Micro Studio. These images were then filtered by selecting the pixels with intensity greater than the mode.

Atomic force microscopy of TiO_2

The surface topography of the TiO_2 samples was also characterized by atomic force microscopy. We found a thickness of 500 nm with a roughness of 100 nm for the TiO_2 nanoparticle film. For these measurements, we used a Park AFM NX20 under ambient conditions. All imaging was acquired in tapping mode using silicon-tip on nitride lever probes (ScanAsyst, f_0 = 70 kHz, Bruker) with a reflective aluminum coating. Atomic force microscopy images were acquired with 256 × 256 pixels and a 0.3 Hz cantilever-tip scan rate. Image analysis was performed using the NanoScope analysis software (version 1.5).

Persson CID model

In this work we used a model proposed by Persson to quantitatively describe the influence of the chemical environment on the SPR of metal particles (*36*). Here we provide a simplified description of this theory, adopting the same terminology and symbolic notations used by Persson in the original publication (*36*).

In this model the width (full width at half maximum, FWHM) γ of the SPR is given by,

$$\gamma = \frac{C v_F}{R} \quad \text{(S.1)}$$

where v_F is the Fermi velocity for a spherical particle with radius *R* embedded in a matrix, and *C* represents the proportionality constant of contributions from diffusive scattering of the electrons at the particle surface and depends on the particle radius (*89*). The tangential and normal components of the adsorbate induced surface electric field gives two contributions to γ, denoted as $\gamma_{\parallel}$ and $\gamma_{\perp}$, respectively. The tangential component $\gamma_{\parallel}$ is given by,

$$\gamma_{\parallel} = \frac{3}{8}\frac{v_F}{R} n_a \sigma_{\text{diff}}(\Omega) \quad \text{(S.2)}$$

where n_a is the number of adsorbates per unit surface area and σ_{diff} is the cross section for diffusive electron scattering evaluated at the SPR frequency Ω and is given by,

$$\sigma_{\text{diff}}(\Omega) = \sigma_0 J(\Omega) = \frac{64}{3\pi}\frac{\omega_F Q}{n v_F} J(\Omega) \quad \text{(S.3)}$$

where ω_F is the Fermi frequency, *n* is the carrier concentration, and *Q* is a number that depends on the symmetry of the resonance state with *Q* = 0.2 for s or p_z symmetry and *Q* = 0.3 for p_x, p_y

symmetry of the adsorbate orbitals. The function σ_{diff} depends on the nature of the chemical bond between the adsorbate molecules and the particle substrate. The integral $J(\Omega)$ is given by,

$$J(\Omega) = \frac{\pi}{4\varepsilon_F}\frac{1}{\hbar\omega}\int_{\varepsilon_F-\hbar\omega}^{\varepsilon_F} d\varepsilon[\varepsilon\Lambda\rho_a(\varepsilon+\hbar\omega)+(\varepsilon+\hbar\omega)\Lambda\rho_a(\varepsilon)] \quad \text{(S.4)}$$

where the projected density of states $\rho_a(\varepsilon)$ is given by,

$$\rho_a = \frac{\Lambda/2}{\pi(\varepsilon-\varepsilon_a)^2+(\Lambda/2)^2} \quad \text{(S.5)}$$

and ε_a is the position of the adsorbate induced resonance or virtual state with width (FWHM) Λ (see Fig. 5 of main text and Fig. 2 of Ref. (*36*)) and $\varepsilon_F = \hbar\omega_F$ is the Fermi energy. The evaluation of $J(\Omega)$ at the observed position of the SPR (Ω) and inserting the obtained value of σ_{diff} in Eq. (S.2) gives the tangential component of the adsorbate induced contribution to the SPR width.

The normal component $\gamma_\perp$ is given by,

$$\gamma_\perp = \frac{16\pi}{1+2\varepsilon_0}\frac{n_a}{R}\Omega \,\text{Im}\,\alpha_\perp(\Omega) \quad \text{(S.6)}$$

where ε_0 is the bulk dielectric constant and α is the polarizability of the adsorbate. The integral Im $\alpha_\perp$ is given by,

$$\text{Im}\,\alpha_\perp(\omega) = 2\pi(ed)^2\int_{\varepsilon_F-\hbar\omega}^{\varepsilon_F} d\varepsilon\rho_a(\varepsilon)\rho_a(\varepsilon+\hbar\omega) \quad \text{(S.7)}$$

where e is the elementary charge and d is the separation of the dynamic image plane and the center of the adsorbate orbital. The evaluation of Im $\alpha_\perp(\Omega)$ at the observed position of the SPR (Ω) by using Eq. (S.7) and inserting the obtained value in Eq. (S.6) gives the normal component of the adsorbate induced contribution to the SPR width.

To calculate the extent of CID for AuNRs@TiO_2 with the Persson model, we used ε_F = 5.53 eV, v_F=1.40×10^{16} Å/s, and n = 0.059 Å^{-3} for Au (*90*). With Q = 0.3 for p_x and p_y orbital symmetry of TiO_2 and introducing an extra factor of two to account for both of the 2π* resonance states in Eq. (S.3), we obtained σ_0=41.4 Å^2. To find the value of ε_a we used the sum of the Au-TiO_2 Schottky barrier and one half of Λ of the TiO_2 induced virtual/resonance state. The Schottky barrier is defined as the energy difference between the valence (or conduction) band edge of the semiconductor and the Fermi energy of the metal (*91*). However, in the Persson model, ε_a is the center of the adsorbate induced resonance state and not its edge. The sum of one half of the width Λ and the Schottky barrier therefore gives the correct value for ε_a. Using a Schottky barrier of 1.25 eV (*54*) and Λ = 0.8 eV for the TiO_2 induced virtual/resonance state centered at $\varepsilon_a - \varepsilon_F$=1.65 eV, Eq. (S.4) was used to evaluate the integral $J(\omega)$ (Fig. S7). The value of $J(\Omega)$ at the observed position of the SPR (Ω=1.82 eV) was calculated to be 0.19 eV.

The Persson model assumes that the metallic particles are spherical objects with radius R. The AuNR@TiO_2 hybrids were rod shaped though with core dimensions of 26 ± 2 × 49 ± 3 nm. We therefore calculated the electron mean free path of the AuNR core by using the geometrical probability approach given by Coronado and Schatz (*80*) and then determined the radius of a sphere with the same mean free path. According to this approach, the effective mean free path L_{eff} of a prolate cylinders is $L_{\text{eff}} = 2d/(r+2)$ with d the diameter and D the height of the cylinder, yielding an aspect ratio $r = d/D$. For the AuNR@TiO_2 sample, we hence employed d = 26 ± 2 nm, D = 49 ± 3 nm, r = 0.53 ± 0.07 nm and L_{eff} = 20.5 ± 1.5 nm. To calculate the radius R of an equivalent sphere with L_{eff} = 20.5 nm, we used $L_{\text{eff}} = (4/3)R$, (*80*), giving R = 15.4 ± 1.1 nm. Inserting this value for R, σ_{diff} (Eq. (S.3)), and the number of TiO_2 resonance states per unit surface area, n_a = 0.1 Å^{-2} (*36*) in Eq. (S.2), we obtained $\gamma_\parallel$=18 ± 1 meV.

To calculate the normal component $\gamma_\perp$, we used the separation of the dynamic image plane and the center of the TiO_2 orbital, $d = D_2 - (D_1/2) = 0.966$ Å where D_1 = 2.04 Å is the

distance between mean planes of two Au layers in the crystal structure of Au (*92*) and D_2 = 1.98 Å is the distance between the mean plane of the outer Au layer and the TiO_2 layer (*93*). Inserting the elementary charge e = 3.794 $eV^{1/2}$ $Å^{1/2}$ and accounting for both 2π* orbitals of TiO_2, Im $\alpha_\perp(\omega)$ at the observed position of the SPR (Ω) was calculated from Eq. (S.7) and is shown in Fig. S8. Inserting the value of Im $\alpha_\perp(\omega)$ and the dielectric constant of TiO_2 (*94*), ε_0 = 60, in Eq. (S.6), we obtained $\gamma_\perp = 1 \pm 0.1$ meV. The total width due to CID,

$$\Gamma_{CID} = \gamma_\perp + \gamma_\parallel, \tag{S.8}$$

is therefore 19 ± 1 meV.

Hypothetical systems in which the broadening arises from isolated defect sites at the surface were also considered. This calculation was achieved by varying both the resonance state energy, ε_a, and the adsorbate concentration, n_a, leaving the rest of the parameters as described above. The results are summarized in Table S1. The total broadening in these cases is far below the experimentally observed broadening, indicating that the broadening arises from states inherent to the interface itself, rather than from isolated defect sites.

Open source software to evaluate the integrals $J(\omega)$ and Im $\alpha_\perp(\omega)$, and hence to calculate $\gamma_\parallel$ and $\gamma_\perp$ may be downloaded at: https://zenodo.org/doi/10.5281/zenodo.11243156
We ask that future users of this tool cite the present paper.

Additional resource for Persson model

Additional open-source software that is subject to future development may be downloaded at: https://github.com/blevine37/ChemDampPersson

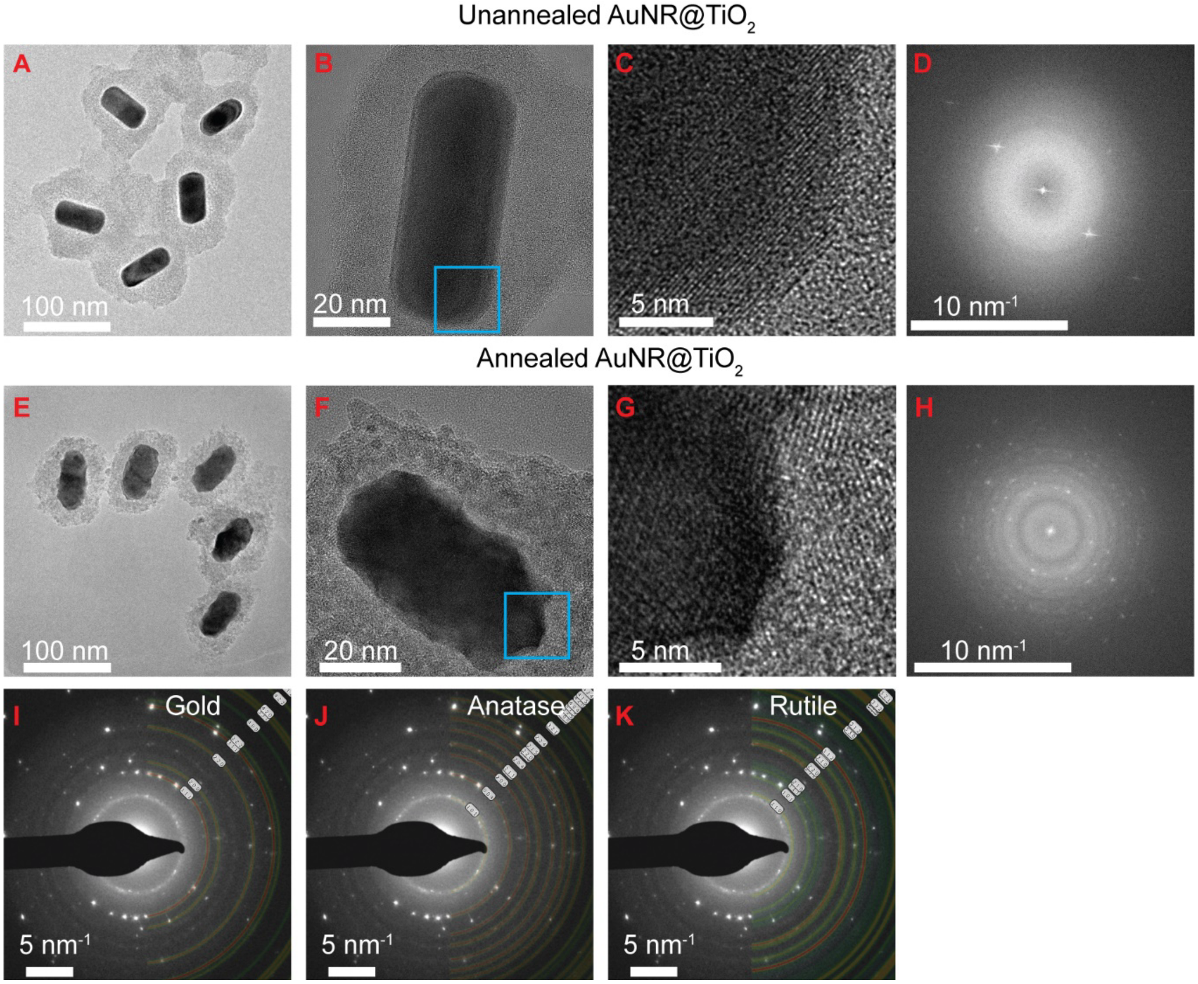

Fig. S1. High resolution transmission electron microscopy of AuNR@TiO_2 hybrids before (A-C) and after thermal annealing (E-G). The unannealed particles exhibited an amorphous TiO_2 shell while the crystalline Au core is apparent (C, D). After thermal annealing, the TiO_2 shell showed domains of crystalline TiO_2. Thermal annealing also slightly reshaped the AuNR core, but overall nanorod geometry was maintained. Although the crystallinity of the core was obfuscated by the crystal lattice of the TiO_2 (G, H), we did not expect a change for Au. We determined that the TiO_2 was in the anatase crystal structure by fitting the diffraction rings in the selected area diffraction pattern to Au (I), anatase (J), and rutile (K) diffraction patterns and found excellent agreement with Au and anatase, but not with rutile.

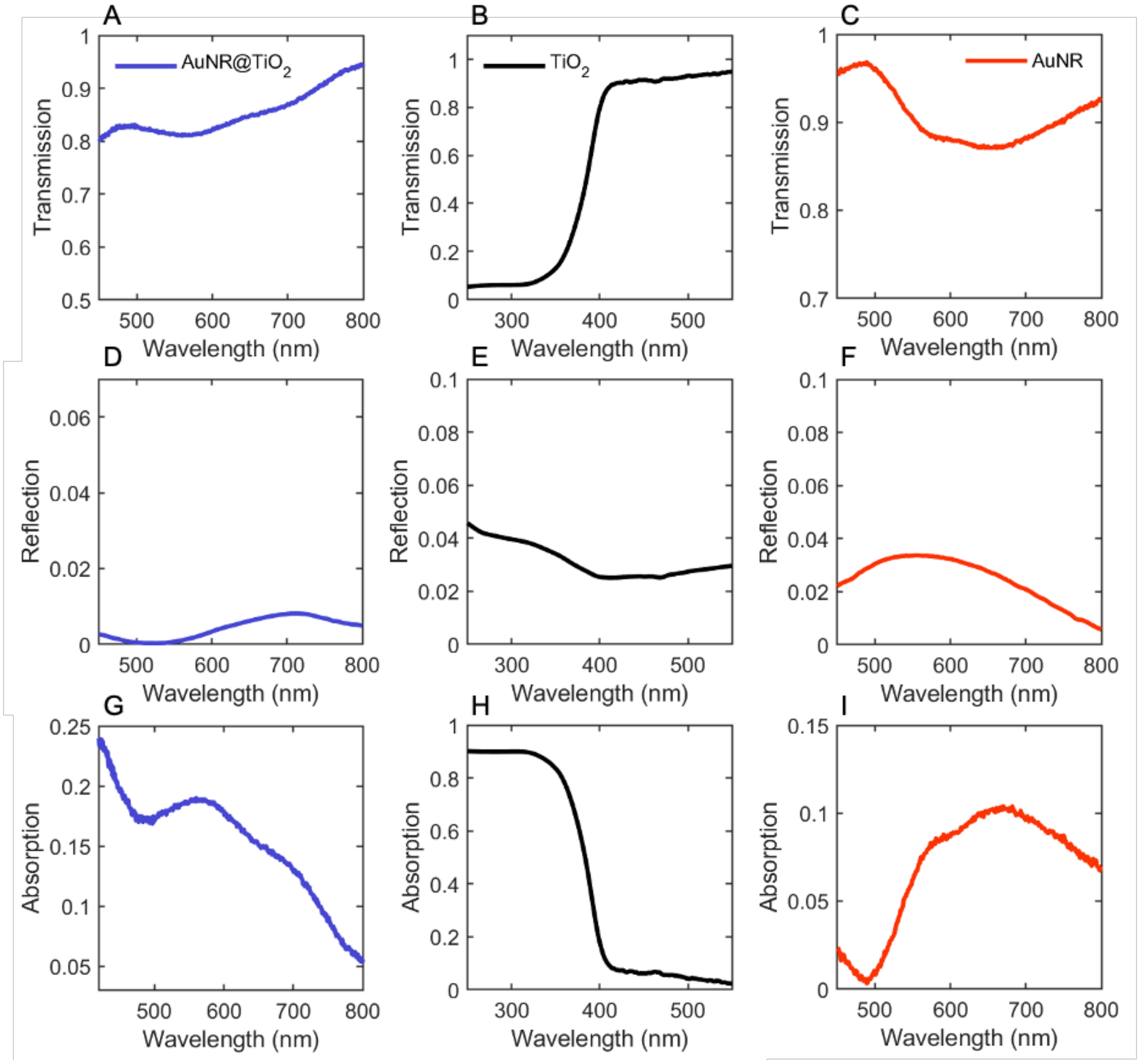

Fig. S2. (A-C) Transmission, (D-F) reflection, and (G-I) absorption of films made from AuNRs@TiO_2 (blue), TiO_2 (black), and AuNRs (red). It is possible that the reported charge carrier injection efficiency at 400 nm was overestimated if TiO_2 can be directly excited. This issue could be a concern especially because the absorption properties of TiO_2 can exhibit variations based on its crystallinity. However, The TiO_2 used here has almost negligible absorption beyond 400 nm (Fig. S2H), different from Ref. (*19*). It is therefore necessary to always emphasize the importance of material-specific characterization due to such variability in absorption characteristics (*88*).

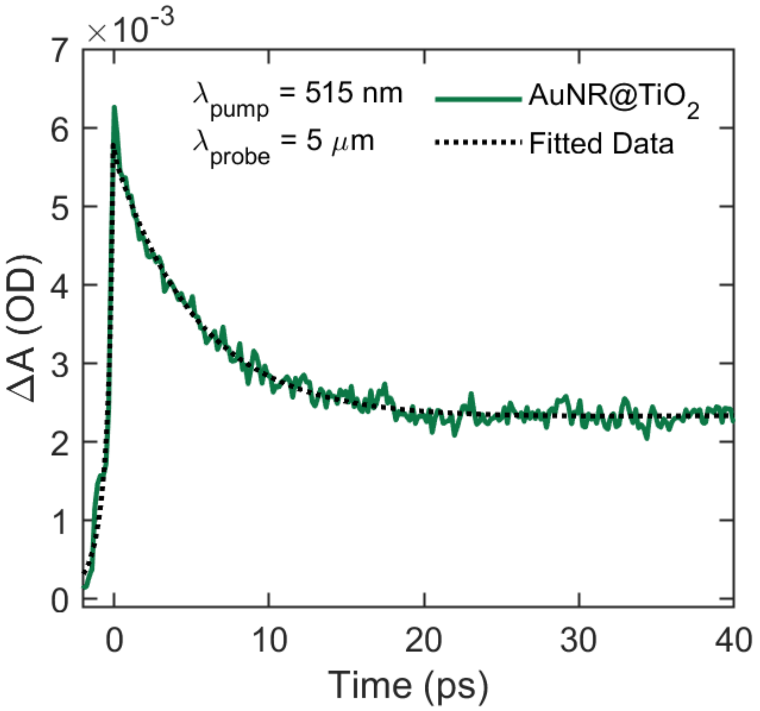

Fig. S3. Ultrafast rise and decay of AuNR@TiO_2 heterostructures probed at 5 μm upon 515 nm excitation (green line). The black dashed line shows the fit to the experimental data, exhibiting an instrument-limited rise time of ~200 fs.

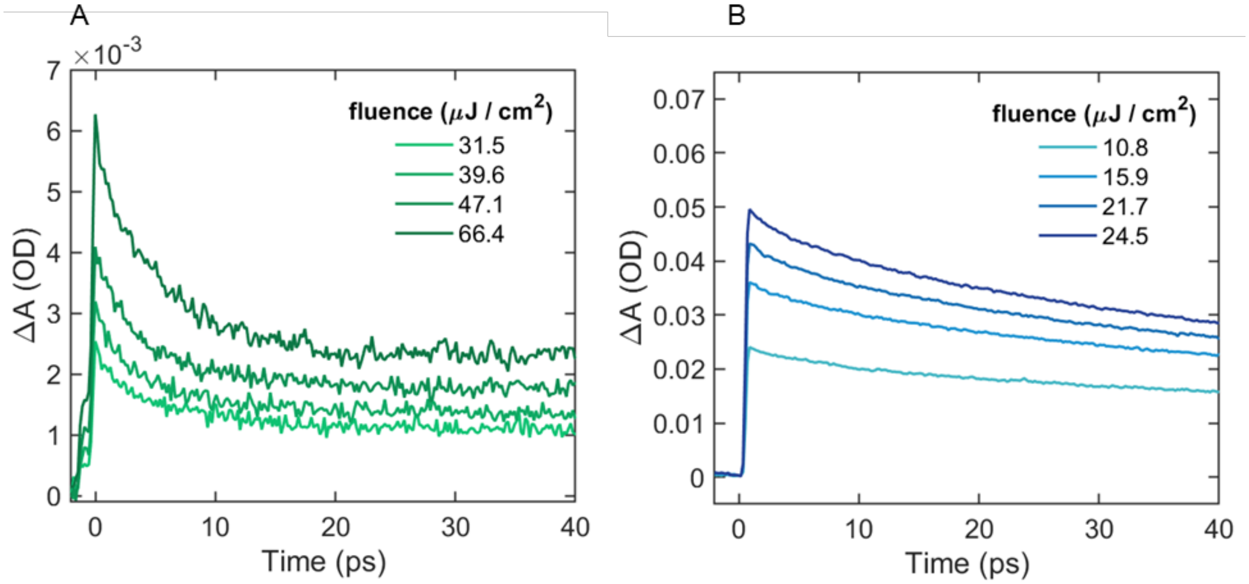

Fig. S4. (A) Fluence-dependent IR transient absorption pump-probe spectroscopy of AuNR@TiO_2 heterostructures. Ultrafast dynamics of AuNR@TiO_2 heterostructures were probed at 5 μm upon 515 nm excitation at various incident fluences, as indicated in the legend. (B) Fluence-dependent IR transient absorption pump-probe spectroscopy of a TiO_2 sample. Ultrafast dynamics of TiO_2 were probed at 5 μm upon 345 nm excitation at various incident fluences, as indicated in the legend.

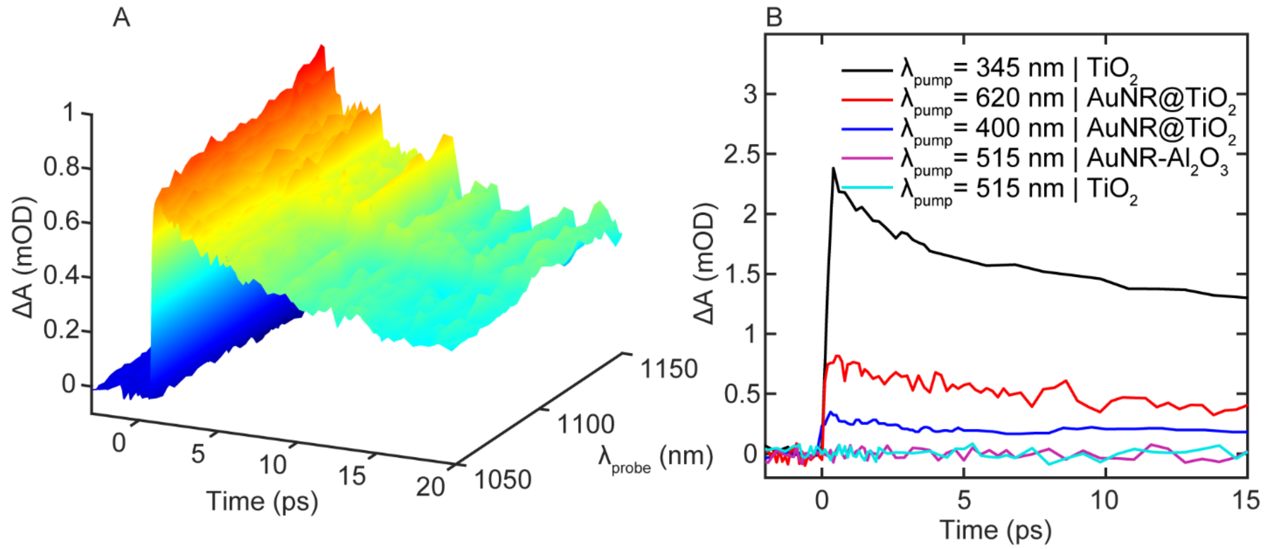

Fig. S5. (A) Ultrafast NIR transient absorption spectra of AuNR@TiO_2 heterostructures probed at 1.05-1.2 µm following 620 nm excitation. (B) Ultrafast rise and decay probed at 1.15 µm for TiO_2 with 266 nm excitation (black), TiO_2 with 515 nm excitation (cyan), AuNRs@TiO_2 with 620 nm excitation (red), AuNRs@TiO_2 with 400 nm excitation (blue), and bare AuNRs with 515 nm excitation (pink).

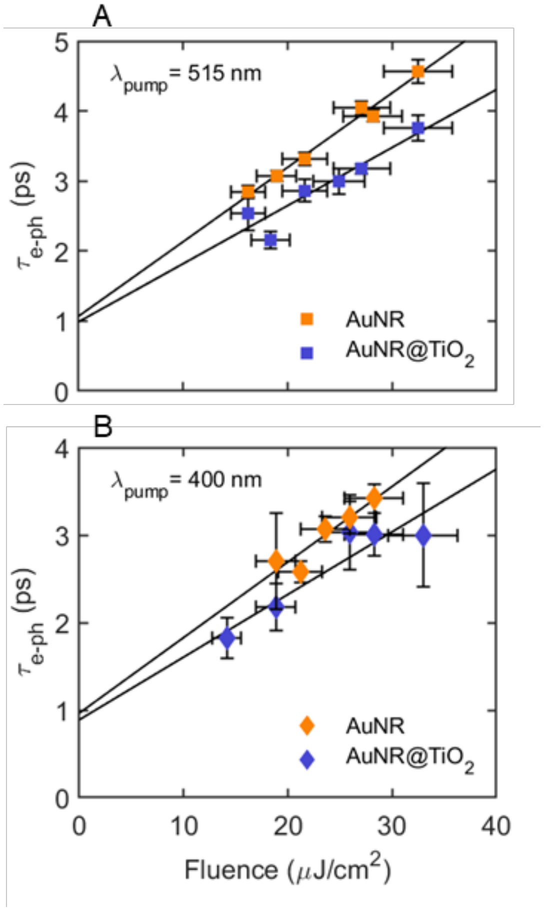

Fig. S6. (A) Electron-phonon relaxation times τ_{e-ph} extracted from fitting the transient absorption traces to the two-temperature model as a function of incident pump fluence for AuNRs (orange squares) and AuNRs@TiO_2 (blue squares) with 515 nm excitation. The probe wavelength was at the SPR maxima of 685 and 715 nm for the AuNR and AuNR@TiO_2 samples, respectively. The lines are linear regressions of the data. (B) Electron-phonon relaxation times τ_{e-ph} extracted from fitting the transient absorption traces to the two-temperature model as a function of incident pump fluence for AuNRs (orange diamonds) and AuNRs@TiO_2 (blue diamonds) with 400 nm excitation. The probe wavelength was at the SPR maxima of 685 and 715 nm for the AuNR and AuNR@TiO2 samples, respectively. The lines are linear regressions of the data. Table S1 lists the fitted slopes.

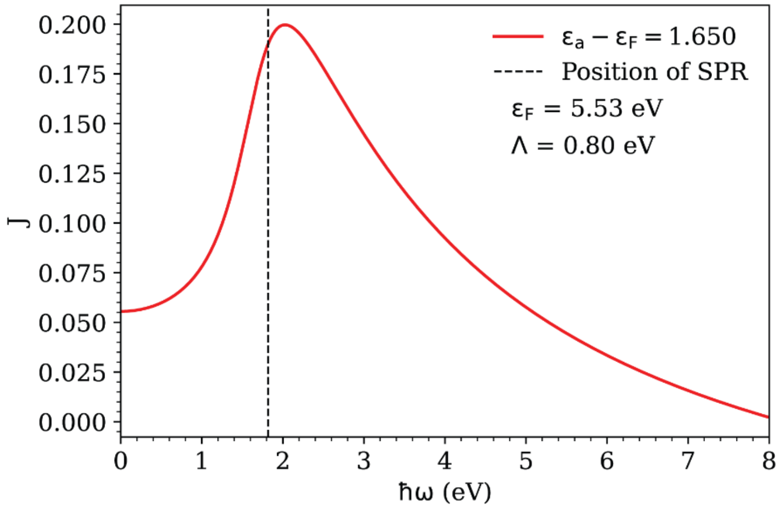

Fig. S7. The integral $J(\omega)$ for the TiO_2 induced resonance state with width (FWHM) $\Lambda = 0.8$ eV. The dotted line represents the observed position (Ω =1.82 eV) of the AuNR SPR.

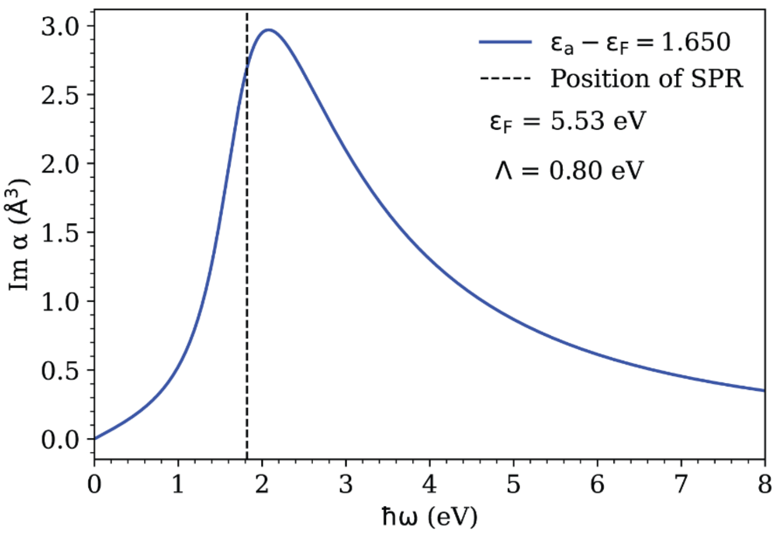

Fig. S8. The integral Im $\alpha_{\perp}(\omega)$ for the TiO_2 induced resonance state with width (FWHM) Λ = 0.8 eV. The dotted line represents the observed position (Ω=1.82 eV) of the AuNR SPR.

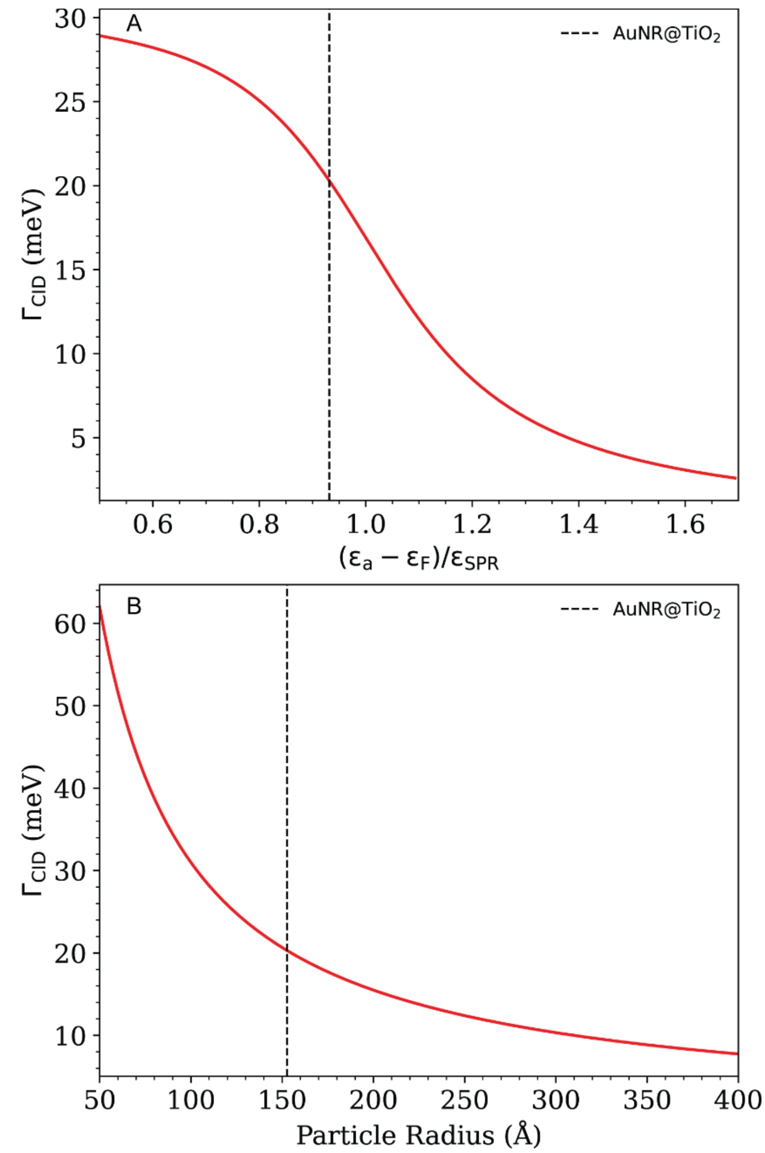

Fig. S9. Effect of adsorbate resonance state and effective particle radius on CID. (A) Change in Γ_{CID} as a function of the ratio of the absorbate resonance and the SPR. As the plasmon energy becomes larger than the adsorbate resonance energy, $(\varepsilon_a - \varepsilon_F)/\varepsilon_{SPR} \rightarrow 0$, charge transfer is predicted to increase. Note that there is no hard cut-off for $(\varepsilon_a - \varepsilon_F)/\varepsilon_{SPR} > 1$ here because of the width Λ of the adsorbate state. (B) Change in Γ_{CID} as a function of equivalent particle radius. Smaller particles are expected to have larger CID.

Figure	Sample	Pump	Probe	Slope
3E	TiO_2	345 nm	5 µm	12.5 ± 0.6 mOD × cm^3/photon ×10^{-18}
3E	AuNRs@TiO_2	515 nm	5 µm	1.9 ± 0.1 mOD × cm^3/photon ×10^{-18}
3E	TiO_2	266 nm	1.15 µm	2.0 ± 0.2 mOD × cm^3/photon ×10^{-18}
3E	AuNRs@TiO_2	515 nm	1.15 µm	0.4 ± 0.1 mOD × cm^3/photon ×10^{-18}
3E	AuNRs@TiO_2	650 nm	1.15 µm	0.9 ± 0.1 mOD × cm^3/photon ×10^{-18}
4C	AuNRs	620 nm	685 nm	130 ± 8 fs × cm^2/µJ
4C	AuNRs@TiO_2	620 nm	715 nm	67 ± 1 fs × cm^2/µJ
S6A	AuNRs	515 nm	685 nm	107 ± 1 fs × cm^2/µJ
S6A	AuNRs@TiO_2	515 nm	715 nm	84 ± 1 fs × cm^2/µJ
S6B	AuNRs	400 nm	685 nm	87 ± 9 fs × cm^2/µJ
S6B	AuNRs@TiO_2	400 nm	715 nm	71 ± 1 fs × cm^2/µJ

Table S1. Fitted slopes of pump power dependent measurements, forcing the intercept to be 0, as discussed in the main text and supplementary information.

ε_a (eV)	n_a (Å^{-2})	$\gamma_\parallel$ (meV)	$\gamma_\perp$ (meV)
7.18	0.01	1.7	0.1
	0.001	0.170	0.01
7.08	0.01	1.9	0.1
	0.001	0.19	0.01
6.93	0.01	2.1	0.2
	0.001	0.21	0.02
6.68	0.01	2.3	0.2
	0.001	0.23	0.02

Table S2. Tangential and normal contributions of TiO_2 to the SPR width calculated using different values of ε_a (eV) at two different surface adsorbate concentrations.

REFERENCES AND NOTES

1. S. Mubeen, J. Lee, N. Singh, S. Kramer, G. D. Stucky, M. Moskovits, An autonomous photosynthetic device in which all charge carriers derive from surface plasmons. *Nat. Nanotechnol*. **8**, 247–251 (2013).

2. M. L. Brongersma, N. J. Halas, P. Nordlander, Plasmon-induced hot carrier science and technology. *Nat. Nanotechnol*. **10**, 25–34 (2015).

3. C. Clavero, Plasmon-induced hot-electron generation at nanoparticle/metal-oxide interfaces for photovoltaic and photocatalytic devices. *Nat. Photonics* **8**, 95–103 (2014).

4. E. Schroeder, P. Christopher, Chemical production using light: Are sustainable photons cheap enough? *ACS Energy Lett*. **7**, 880–884 (2022).

5. K. A. Willets, R. P. Van Duyne, Localized surface plasmon resonance spectroscopy and sensing. *Annu. Rev. Phys. Chem.* **58**, 267–297 (2007).

6. G. V. Hartland, Optical studies of dynamics in noble metal nanostructures. *Chem. Rev.* **111**, 3858–3887 (2011).

7. S. Link, M. A. El-Sayed, Shape and size dependence of radiative, non-radiative and photothermal properties of gold nanocrystals. *Int. Rev. Phys. Chem*. **19**, 409–453 (2000).

8. J. B. Khurgin, How to deal with the loss in plasmonics and metamaterials. *Nat. Nanotechnol*. **10**, 2–6 (2015).

9. D. C. Ratchford, Plasmon-induced charge transfer: Challenges and outlook. *ACS Nano* **13**, 13610–13614 (2019).

10. S. K. Giri, G. C. Schatz, Photodissociation of H2 on Ag and Au nanoparticles: Effect of size and plasmon versus interband transitions on threshold intensities for dissociation. *J. Phys. Chem. C* **127**, 4115–4123 (2023).

11. C. L. Warkentin, R. R. Frontiera, Quantifying the ultrafast and steady-state molecular reduction potential of a plasmonic photocatalyst. *Proc. Natl. Acad. Sci. U.S.A.* **120**, e2305932120 (2023).

12. G. Tagliabue, J. S. DuChene, M. Abdellah, A. Habib, D. J. Gosztola, Y. Hattori, W.-H. Cheng, K. Zheng, S. E. Canton, R. Sundararaman, J. Sa, H. A. Atwater, Ultrafast hothole

injection modifies hot-electron dynamics in Au/p-GaN heterostructures. *Nat. Mater.* **19**, 1312–1318 (2020).

13. D. C. Ratchford, A. D. Dunkelberger, I. Vurgaftman, J. C. Owrutsky, P. E. Pehrsson, Quantification of efficient plasmonic hot-electron injection in gold nanoparticle–TiO2 *films. Nano Lett*. **17**, 6047–6055 (2017).
14. P. Christopher, M. Moskovits, Hot charge carrier transmission from plasmonic nanostructures. *Annu. Rev. Phys. Chem.* **68**, 379–398 (2017).
15. K. Wu, J. Chen, J. R. McBride, T. Lian, Efficient hot-electron transfer by a plasmoninduced interfacial charge-transfer transition. *Science* **349**, 632–635 (2015).
16. L. Du, A. Furube, K. Hara, R. Katoh, M. Tachiya, Ultrafast plasmon induced electron injection mechanism in gold–TiO2 nanoparticle system. *J. Photochem. Photobiol. C Photochem. Rev.* **15**, 21–30 (2013).
17. A. Furube, L. Du, K. Hara, R. Katoh, M. Tachiya, Ultrafast plasmon-induced electron transfer from gold nanodots into TiO2 nanoparticles. *J. Am. Chem. Soc.* **129**, 14852–14853 (2007).
18. L. Du, X. Shi, G. Zhang, A. Furube, Plasmon induced charge transfer mechanism in gold-TiO2 nanoparticle systems: The size effect of gold nanoparticle. *J. Appl. Phys*. **128**, 213104 (2020).
19. J. Song, J. Long, Y. Liu, Z. Xu, A. Ge, B. D. Piercy, D. A. Cullen, I. N. Ivanov, J. R. McBride, M. D. Losego, T. Lian, Highly efficient plasmon induced hot-electron transfer at Ag/TiO2 Interface. *ACS Photonics* **8**, 1497–1504 (2021).
20. B. Doiron, Y. Li, R. Bower, A. Mihai, S. Dal Forno, S. Fearn, L. Huttenhofer, E. Cortes, L. F. Cohen, N. M. Alford, J. Lischner, P. Petrov, S. A. Maier, R. F. Oulton, Optimizing hot electron harvesting at planar metal–Semiconductor interfaces with titanium oxynitride thin films. *ACS Appl. Mater. Interfaces* **15**, 30417–30426 (2023).
21. L. V. Melendez, J. Van Embden, T. U. Connell, N. W. Duffy, D. E. Gomez, Optimal geometry for plasmonic hot-carrier extraction in metal–Semiconductor nanocrystals. *ACS Nano* **17**, 4659–4666 (2023).

22. Y. Tian, T. Tatsuma, Mechanisms and applications of plasmon-induced charge separation at TiO2 films loaded with gold nanoparticles. *J. Am. Chem. Soc.* **127**, 7632–7637 (2005).

23. J. S. DuChene, B. C. Sweeny, A. C. Johnston-Peck, D. Su, E. A. Stach, W. D. Wei, Prolonged hot electron dynamics in plasmonic-metal/semiconductor heterostructures with implications for solar photocatalysis. *Angew. Chem. Int. Ed. Engl* **53**, 7887–7891 (2014).

24. J. Sa, G. Tagliabue, P. Friedli, J. Szlachetko, M. H. Rittmann-Frank, F. G. Santomauro, C. J. Milne, H. Sigg, Direct observation of charge separation on Au localized surface plasmons. *Energy Environ. Sci*. **6**, 3584 (2013).

25. A. L. Linsebigler, G. Lu, J. T. Yates Jr., Photocatalysis on TiO2 surfaces: Principles, mechanisms, and selected results. *Chem. Rev*. **95**, 735–758 (1995).

26. Y. Zhang, S. He, W. Guo, Y. Hu, J. Huang, J. R. Mulcahy, W. D. Wei, Surface-plasmondriven hot electron photochemistry. *Chem. Rev*. **118**, 2927–2954 (2018).

27. V. Subramanian, E. Wolf, P. V. Kamat, Semiconductor − metal composite nanostructures. To what extent do metal nanoparticles improve the photocatalytic activity of TiO2 films? *J. Phys. Chem. B* **105**, 11439–11446 (2001).

28. C. Ng, J. J. Cadusch, S. Dligatch, A. Roberts, T. J. Davis, P. Mulvaney, D. E. Gomez, Hot carrier extraction with plasmonic broadband absorbers. *ACS Nano* **10**, 4704–4711 (2016).

29. T. Ishida, T. Tatsuma, Effect of plasmon coupling on quantum efficiencies of plasmoninduced charge separation. *J. Phys. Chem. C* **122**, 26153–26159 (2018).

30. F. Kiani, A. R. Bowman, M. Sabzehparvar, C. O. Karaman, R. Sundararaman, G. Tagliabue, Transport and interfacial injection of d-band hot holes control plasmonic chemistry. *ACS Energy Lett.* **8**, 4242–4250 (2023).

31. X. Shi, X. Li, T. Toda, T. Oshikiri, K. Ueno, K. Suzuki, K. Murakoshi, H. Misawa, Interfacial structure-modulated plasmon-induced water oxidation on strontium titanate.

ACS Appl. Energy Mater. **3**, 5675–5683 (2020).

32. A. O. Govorov, H. Zhang, Y. K. Gun′ko, Theory of photoinjection of hot plasmonic carriers from metal nanostructures into semiconductors and surface molecules. *J. Phys. Chem. C* **117**, 16616–16631 (2013).

33. C. Boerigter, R. Campana, M. Morabito, S. Linic, Evidence and implications of direct charge excitation as the dominant mechanism in plasmon-mediated photocatalysis. *Nat. Commun.* **7**, 10545 (2016).

34. S. Tan, A. Argondizzo, J. Ren, L. Liu, J. Zhao, H. Petek, Plasmonic coupling at a metal/semiconductor interface. *Nat. Photonics* **11**, 806–812 (2017).

35. H. Hovel, S. Fritz, A. Hilger, U. Kreibig, M. Vollmer, Width of cluster plasmon resonances: Bulk dielectric functions and chemical interface damping. *Phys. Rev. B* **48**, 18178–18188 (1993).

36. B. N. J. Persson, Polarizability of small spherical metal particles: Influence of the matrix environment. *Surf. Sci*. **281**, 153–162 (1993).

37. J. Ma, S. Gao, Plasmon-induced electron–Hole separation at the Ag/TiO2(110) interface. *ACS Nano* **13**, 13658–13667 (2019).

38. H. Petek, S. Ogawa, Femtosecond time-resolved two-photon photoemission studies of electron dynamics in metals. *Prog. Surf. Sci.* **56**, 239–310 (1997).

39. J. Cao, Y. Gao, H. E. Elsayed-Ali, R. J. D. Miller, D. A. Mantell, Femtosecond photoemission study of ultrafast electron dynamics in single-crystal Au(111) films. *Phys. Rev. B* **58**, 10948–10952 (1998).

40. C.-K. Sun, F. Vallee, L. H. Acioli, E. P. Ippen, J. G. Fujimoto, Femtosecond-tunable measurement of electron thermalization in gold. *Phys. Rev. B* **50**, 15337–15348 (1994).

41. H. Ahmad, S. K. Kamarudin, L. J. Minggu, M. Kassim, Hydrogen from photo-catalytic

water splitting process: A review. *Renew. Sustain. Energy Rev.* **43**, 599–610 (2015).

42. K. Hashimoto, H. Irie, A. Fujishima, TiO_2 photocatalysis: A historical overview and future prospects. *Jpn. J. Appl. Phys.* **44**, 8269–8285 (2005).

43. Y. Li, J. G. DiStefano, A. A. Murthy, J. D. Cain, E. D. Hanson, Q. Li, F. C. Castro, X. Chen, V. P. Dravid, Superior plasmonic photodetectors based on Au@MoS_2 core–Shell heterostructures. *ACS Nano* **11**, 10321–10329 (2017).

44. C. Fang, H. Jia, S. Chang, Q. Ruan, P. Wang, T. Chen, J. Wang, (Gold core)/(titania shell) nanostructures for plasmon-enhanced photon harvesting and generation of reactive oxygen species. *Energy Environ. Sci.* **7**, 3431–3438 (2014).

45. C. Hu, X. Zhang, X. Li, Y. Yan, G. Xi, H. Yang, H. Bai, Au photosensitized TiO_2 ultrathin nanosheets with {001} exposed facets. *Chem. A Eur. J.* **20**, 13557–13560 (2014).

46. A. Sousa-Castillo, M. Comesana-Hermo, B. Rodriguez-Gonzalez, M. Perez-Lorenzo, Z. Wang, X.-T. Kong, A. O. Govorov, M. A. Correa-Duarte, Boosting hot electron-driven photocatalysis through anisotropic plasmonic nanoparticles with hot spots in Au–TiO_2 nanoarchitectures. *J. Phys. Chem. C* **120**, 11690–11699 (2016).

47. B. Ostovar, Y.-Y. Cai, L. J. Tauzin, S. A. Lee, A. Ahmadivand, R. Zhang, P. Nordlander, S. Link, Increased intraband transitions in smaller gold nanorods enhance light emission. *ACS Nano* **14**, 15757–15765 (2020).

48 C. Sonnichsen, T. Franzl, T. Wilk, G. von Plessen, J. Feldmann, O. Wilson, P. Mulvaney, Drastic reduction of plasmon damping in gold nanorods. *Phys. Rev. Lett.* **88**, 077402 (2002).

49. P. K. Jain, W. Huang, M. A. El-Sayed, On the universal scaling behavior of the distance decay of plasmon coupling in metal nanoparticle pairs: A plasmon ruler equation. *Nano Lett.* **7**, 2080–2088 (2007).

50. J. Olson, S. Dominguez-Medina, A. Hoggard, L.-Y. Wang, W.-S. Chang, S. Link, Optical

characterization of single plasmonic nanoparticles. *Chem. Soc. Rev*. **44**, 40–57 (2015).

51. B. Foerster, M. Hartelt, S. S. E. Collins, M. Aeschlimann, S. Link, C. Sonnichsen, Interfacial states cause equal decay of plasmons and hot electrons at gold–Metal oxide interfaces. *Nano Lett*. **20**, 3338–3343 (2020).

52. M. Hu, C. Novo, A. Funston, H. Wang, H. Staleva, S. Zou, P. Mulvaney, Y. Xia, G. V. Hartland, Dark-field microscopy studies of single metal nanoparticles: Understanding the factors that influence the linewidth of the localized surface plasmon resonance. *J. Mater. Chem.* **18**, 1949–1960 (2008).

53. U. Kreibig, M. Vollmer, *Optical Properties of Metal Clusters* (Springer, 1995) vol. **25**; htt p://link.springer.com/10.1007/978-3-662-09109-8.

54 S. A. Lee, S. Link, Chemical interface damping of surface plasmon resonances. Acc. Chem. Res. 54, 1950–1960 (2021).

55. S. A. Lee, B. Ostovar, C. F. Landes, S. Link, Spectroscopic signatures of plasmoninduced charge transfer in gold nanorods. *J. Chem. Phys.* **156**, 064702 (2022).

56. B. Foerster, A. Joplin, K. Kaefer, S. Celiksoy, S. Link, C. Sonnichsen, Chemical interface damping depends on electrons reaching the surface. *ACS Nano* **11**, 2886–2893 (2017).

57. R. L. Olmon, B. Slovick, T. W. Johnson, D. Shelton, S.-H. Oh, G. D. Boreman, M. B. Raschke, Optical dielectric function of gold. *Phys. Rev. B* **86**, 235147 (2012).

58. A. Hoggard, L.-Y. Wang, L. Ma, Y. Fang, G. You, J. Olson, Z. Liu, W.-S. Chang, P. M. Ajayan, S. Link, Using the plasmon linewidth to calculate the time and efficiency of electron transfer between gold nanorods and graphene. *ACS Nano* **7**, 11209–11217 (2013).

59. J. J. Mock, R. T. Hill, A. Degiron, S. Zauscher, A. Chilkoti, D. R. Smith, Distancedependent plasmon resonant coupling between a gold nanoparticle and gold film. *Nano Lett.* **8**, 2245–2252 (2008).

60. T. Yoshihara, R. Katoh, A. Furube, Y. Tamaki, M. Murai, K. Hara, S. Murata, H.

Arakawa, M. Tachiya, Identification of reactive species in photoexcited nanocrystalline TiO2 films by wide-wavelength-Range (400－2500 nm) transient absorption spectroscopy. *J. Phys. Chem. B* **108**, 3817–3823 (2004).

61. H. Jin, J. M. Kahk, D. A. Papaconstantopoulos, A. Ferreira, J. Lischner, Plasmon-induced hot carriers from interband and intraband transitions in large noble metal nanoparticles. *PRX Energy* **1**, 013006 (2022).

62. M. Bauer, A. Marienfeld, M. Aeschlimann, Hot electron lifetimes in metals probed by time-resolved two-photon photoemission. *Prog. Surf. Sci.* **90**, 319–376 (2015).

63. J. Robertson, Band offsets, Schottky barrier heights, and their effects on electronic devices. *J. Vac. Sci. Technol. A* **31**, 050821 (2013).

64. S. Link, M. A. El-Sayed, Spectral properties and relaxation dynamics of surface plasmon electronic oscillations in gold and silver nanodots and nanorods. *J. Phys. Chem. B* **103**, 8410–8426 (1999).

65. J. H. Hodak, A. Henglein, G. V. Hartland, Electron-phonon coupling dynamics in very small (between 2 and 8 nm diameter) Au nanoparticles. *J. Chem. Phys.* **112**, 5942–5947 (2000).

66. C. Voisin, N. Del Fatti, D. Christofilos, F. Vallee, Ultrafast electron dynamics and optical nonlinearities in metal nanoparticles. *J. Phys. Chem. B* **105**, 2264–2280 (2001).

67. A. M. Brown, R. Sundararaman, P. Narang, W. A. Goddard, H. A. Atwater, Ab initio phonon coupling and optical response of hot electrons in plasmonic metals. *Phys. Rev. B* **94**, 075120 (2016).

68. J. H. Hodak, A. Henglein, G. V. Hartland, Size dependent properties of Au particles: Coherent excitation and dephasing of acoustic vibrational modes. *J. Chem. Phys*. **111**, 8613–8621 (1999).

69. J. Hodak, I. Martini, G. V. Hartland, Ultrafast study of electron–phonon coupling in

colloidal gold particles. *Chem. Phys. Lett*. **284**, 135–141 (1998).

70. R. H. M. Groeneveld, R. Sprik, A. Lagendijk, Femtosecond spectroscopy of electronelectron and electron-phonon energy relaxation in Ag and Au. *Phys. Rev. B* **51**, 11433–11445 (2005).

71. W.-Y. Chiang, A. Bruncz, B. Ostovar, E. K. Searles, S. Brasel, G. Hartland, S. Link, Electron-phonon relaxation dynamics of hot electrons in gold nanoparticles are independent of excitation pathway. *J. Phys. Chem. C* **127,** 21176–21185 (2023).

72. A. Arbouet, C. Voisin, D. Christofilos, P. Langot, N. Del Fatti, F. Vallee, J. Lerme, G. Celep, E. Cottancin, M. Gaudry, M. Pellarin, M. Broyer, M. Maillard, M. P. Pileni, M. Treguer, Electron-phonon scattering in metal clusters. *Phys. Rev. Lett*. **90**, 177401 (2003).

73. S. Link, M. A. El-Sayed, Optical properties and ultrafast dynamics of metallic nanocrystals. Annu. *Rev. Phys. Chem*. **54**, 331–366 (2003).

74. S. A. Lee, C. T. Kuhs, E. K. Searles, H. O. Everitt, C. F. Landes, S. Link, *d*-band hole dynamics in gold nanoparticles measured with time-resolved emission upconversion microscopy. *Nano Lett.* **23**, 3501–3506 (2023).

75. M. Bernardi, J. Mustafa, J. B. Neaton, S. G. Louie, Theory and computation of hot carriers generated by surface plasmon polaritons in noble metals. *Nat. Commun*. **6**, 7044 (2015).

76. A. M. Brown, R. Sundararaman, P. Narang, W. A. I. Goddard, H. A. Atwater, Nonradiative plasmon decay and hot carrier dynamics: Effects of phonons, surfaces, and geometry. *ACS Nano* **10**, 957–966 (2016).

77. D. O. Scanlon, C. W. Dunnill, J. Buckeridge, S. A. Shevlin, A. J. Logsdail, S. M. Woodley, C. R. A. Catlow, M. J. Powell, R. G. Palgrave, I. P. Parkin, G. W. Watson, T. W. Keal, P. Sherwood, A. Walsh, A. A. Sokol, Band alignment of rutile and anatase TiO2. *Nat. Mater*. **12,** 798–801 (2013).

78. X. Ye, C. Zheng, J. Chen, Y. Gao, C. B. Murray, Using binary surfactant mixtures to

simultaneously improve the dimensional tunability and monodispersity in the seeded growth of gold nanorods. *Nano Lett.* **13**, 765–771 (2013).

79. B. Wu, D. Liu, S. Mubeen, T. T. Chuong, M. Moskovits, G. D. Stucky, Anisotropic growth of TiO2 onto gold nanorods for plasmon-enhanced hydrogen production from water reduction. *J. Am. Chem. Soc.* **138**, 1114–1117 (2016).

80. E. A. Coronado, G. C. Schatz, Surface plasmon broadening for arbitrary shape nanoparticles: A geometrical probability approach. *J. Chem. Phys.* **119**, 3926–3934 (2003).

81. Y. Fang, W.-S. Chang, B. Willingham, P. Swanglap, S. Dominguez-Medina, S. Link, Plasmon emission quantum yield of single gold nanorods as a function of aspect ratio. *ACS Nano* **6**, 7177–7184 (2012).

82. Y. Yang, H. Xie, J. You, W. Ye, Revisiting the plasmon radiation damping of gold nanorods. *Phys. Chem. Chem. Phys*. **24**, 4131–4135 (2022).

83. J. B. Asbury, N. A. Anderson, E. Hao, X. Ai, T. Lian, Parameters affecting electron injection dynamics from ruthenium dyes to titanium dioxide nanocrystalline thin film. J. *Phys. Chem. B* **107**, 7376–7386 (2003).

84. C.-K. Sun, F. Vallee, L. Acioli, E. P. Ippen, J. G. Fujimoto, Femtosecond investigation of electron thermalization in gold. *Phys. Rev. B* **48**, 12365–12368 (2010).

85. S. I. Anisimov, B. L. Kapeliovich, T. L. Perelʹman, Electron emission from metal surfaces exposed to ultrashort laser pulses. *Zhurnal Eksp. Teor. Fiz*. **66**, 776–781 (1974).

86. Z. Lin, L. V. Zhigilei, V. Celli, Electron-phonon coupling and electron heat capacity of metals under conditions of strong electron-phonon nonequilibrium. *Phys. Rev. B* **77**, 075133 (2008).

87. J. H. Hodak, I. Martini, G. V. Hartland, Spectroscopy and dynamics of nanometer-sized noble metal particles. *J. Phys. Chem. B* **102**, 6958–6967 (1998).

88. T. Luttrell, S. Halpegamage, J. Tao, A. Kramer, E. Sutter, M. Batzill, Why is anatase a better photocatalyst than rutile? - Model studies on epitaxial TiO2 films. *Sci. Rep*. **4**, 4043 (2014).

89. B. N. J. Persson, K. Zhao, Z. Zhang, Chemical contribution to surface-enhanced raman scattering. *Phys. Rev. Lett*. **96,** 207401 (2006).

90. D. R. Lide, *CRC Handbook of Chemistry and Physics* (CRC Press LLC, ed. 85, 2004).

91. P. Bhattacharya, R. Fornari, H. Kamimura, Eds., *Comprehensive Semiconductor Science and Technology* (Elsevier, ed. 1, 2011).

92. X.-s. Yan, P. Lin, X. Qi, L. Yang, Finnis-Sinclair potentials for fcc Au-Pd and Ag-Pt alloys. *Int. J. Mater. Res*. **102**, 381–388 (2011).

93. K. Sun, M. Kohyama, S. Tanaka, S. Takeda, Reaction mechanism of the low-temperature water–Gas shift reaction on Au/TiO2 catalysts. *J. Phys. Chem. C* **121**, 12178–12187 (2017).

94. M. D. Stamate, Dielectric properties of TiO2 thin films deposited by a DC magnetron sputtering system. *Thin Solid Films* **372**, 246–249 (2000).
